# Prevalence of mental disorders among young people living with HIV: a systematic review and meta-analysis

**DOI:** 10.3389/fpubh.2024.1392872

**Published:** 2024-08-21

**Authors:** Shenao Zhan, Fei Ouyang, Wenjing Zhai, Haitao Yang

**Affiliations:** ^1^Key Laboratory of Environmental Medicine Engineering of Ministry of Education, Department of Epidemiology and Health Statistics, School of Public Health, Southeast University, Nanjing, China; ^2^Jiangsu Provincial Center for Disease Control and Prevention, Nanjing, China

**Keywords:** young people, HIV, depression, anxiety, mental disorders, meta-analysis

## Abstract

**Objective:**

This meta-analysis aims to evaluate the global prevalence of mental disorders among young people living with HIV.

**Methods:**

A comprehensive search was conducted of the PubMed, Embase, and Cochrane Library databases for articles relevant to the study, published between January 2013 and June 2023. To identify sources of heterogeneity and compare prevalence estimates among various groups, subgroup analyses were conducted. Study heterogeneity was assessed using Cochran’s Q and the *I*^2^ tests. The robustness of the findings was ascertained through sensitivity analyses, while publication bias was evaluated with funnel plots and Egger’s test.

**Results:**

Sixty studies were included in this meta-analysis. It revealed that approximately one-quarter of YLWH experience depression, with a prevalence of 24.6% (95% CI: 21.1–28.2%). The prevalence of anxiety was found to be 17.0% (95% CI: 11.4–22.6%). Regarding suicidality, the prevalence of suicidal ideation and lifetime suicidal ideation in YLWH was 16.8% (95% CI: 11.3–22.4%) and 29.7% (95% CI: 23.7–35.7%), respectively. Additionally, the prevalence rates for suicidal attempts and lifetime suicidal attempts were 9.7% (95% CI: 4.0–15.4%) and 12.9% (95% CI: 2.8–23.1%), respectively. The prevalence of Post-Traumatic Stress Disorder and Attention Deficit Hyperactivity Disorder was identified as 10.5% (95% CI: 5.8–15.2%) and 5.0% (95% CI: 3.1–7.0%), respectively.

**Conclusion:**

The findings indicate a heightened risk of mental disorders among YLWH, underscoring the necessity for targeted intervention strategies to mitigate their suffering and potentially diminish the adverse impacts.

**Systematic Review Registration:**

PROSPERO, identifier CRD42023470050, https://www.crd.york.ac.uk/prospero/display_record.php?ID=CRD42023470050.

## Introduction

According to the World Health Organization (WHO), individuals aged 10–24 years constitute the young demographic (1). HIV persists as a major global health challenge, affecting approximately 39.0 million people worldwide in 2022, with a significant concentration in the WHO African Region (2). Alarmingly, the incidence of HIV is increasingly prevalent among young people, as evidenced by 480,000 new cases in this age group in 2022 alone (3).

Contemporary research indicates that individuals with HIV are more prone to mental health disorders than their non-infected counterparts (4, 5), a trend also observed in young people living with HIV (YLWH). Depression and anxiety are particularly prevalent mental health issues among YLWH. Research also indicates a significant prevalence of suicide, Post-Traumatic Stress Disorder (PTSD), and Attention-deficit hyperactivity disorder (ADHD) within this demographic (6, 7). The past decade has seen an escalation in research focusing on the mental health of YLWH. However, the global variation in reported mental disorder rates among YLWH underscores the need for more systematic research and thorough analysis. For example, the prevalence of anxiety among YLWH ranges from 2.2% in Indonesia to 56.7% in South Africa (8, 9), and for depression, it ranges from 3.3 to 52.6%, with both studies conducted in Kenya (10, 11). This variance can be attributed to factors such as diverse study population characteristics, disease stages, geographical locations, and the use of different standardized measurement tools. Additionally, research has linked mental health disorders with suboptimal adherence to antiretroviral therapy (ART), resulting in poor virologic control, drug resistance, and heightened HIV morbidity and mortality (12, 13). Therefore, comprehending the worldwide prevalence of mental disorders among young people living with HIV is of paramount importance and urgency.

Prior systematic reviews and meta-analyses have primarily focused on adolescents, with less emphasis on young adults. Our systematic review and meta-analysis extend the age range to encompass YLWH aged 10–24. This study is, to the best of our knowledge, the first to offer a comprehensive global synthesis of data regarding the prevalence of mental disorders among this demographic. The findings will offer critical insights into the prevalence of mental health conditions within this group, thus facilitating the creation of timely and impactful interventions.

## Methods

The Preferred Reporting Items for Systematic reviews and Meta-Analyses (PRISMA) guideline was used to guide this review’s design and reporting (14). Registration of the study was completed under the identifier CRD42023470050 with the International Prospective Register of Systematic Reviews (PROSPERO).

### Search strategy and study eligibility

We conducted searches in PubMed, Embase, and the Cochrane Library from June 20, 2023, to June 30, 2023, for cross-sectional studies, longitudinal studies, and case–control studies on the prevalence of depression, anxiety, suicidality, PTSD, and ADHD YLWH aged 10 to 24 years, published between January 2013 and June 2023. The keywords and their combinations include HIV and all the synonyms; mental disorders, depression, anxiety, suicide, PTSD, ADHD and all their synonyms; adolescents and young people and all the synonyms (The full search strategy see Supplementary material 2 Table S1). The retrieved papers were included in this review if they met the following inclusion and exclusion criteria.

Inclusion criteria: (1) The study population was YLWH aged 10–24 years old; (2) Provide data on the prevalence of any or multiple mental disorders among the following: depression, anxiety, suicidality, PTSD, and ADHD. (3) Cross-sectional studies, longitudinal studies and case–control studies.

Exclusion criteria: (1) Case studies, reviews, comments, conference abstracts, case reports and letters; (2) Not English articles; (3) No data on the prevalence of any of the mental disorders including depression, anxiety, suicidality, PTSD, and ADHD were provided; (4) Age range of the study population is not specified; (5) The study population is not YLWH aged 10–24 years old.

### Screening and data extraction

Following the search strategy, the retrieved articles were imported into Endnote X9, and duplicates were removed. Two researchers (SZ and WZ) independently screened the titles and abstracts for initial selection and then independently read the full texts for secondary screening. Studies to be included were determined based on the inclusion and exclusion criteria. Any discrepancies were adjudicated by a third researcher (FO).

Data extraction was conducted independently by two researchers (SZ and WZ), encompassing various study parameters: (1) Article’s first author and publication year; (2) Country; (3) Study design; (4) ART status; (5) Sample size; (6) Male ratio; (7) Age range; (8) Mean or median age of participants; (9) Period of survey execution; (10) Instruments used for measurement; (11) Cut-off score; (12) Validation information for the measurement tools; (13) Prevalence of depression, anxiety, suicidality, PTSD, and ADHD. Specifically for suicide, separate assessments were made for suicidal ideation, lifetime suicidal ideation, suicidal attempts, and lifetime suicidal attempts. In the case of longitudinal studies, only baseline data were considered.

### Quality assessment

For quality assessment, the Agency for Healthcare Research and Quality (AHRQ) cross-sectional study quality evaluation list, comprising 11 items, was utilized (15). Each item rated “yes” referring value “1” was summed giving a range of a possible total score between 0 and 11 on the checklist. Studies were classified based on quality into three categories: low (0–3 points), medium (4–7 points), and high (8–11 points). The evaluation was conducted by two researchers (SZ and WZ), using the AHRQ criteria. In cases of inconsistent evaluations, a third reviewer (FO) was consulted for reassessment.

### Data analysis

We employed the R4.2.3 software for calculating the aggregated prevalence of various mental disorders. The fixed effect model was applied in cases where the *I*^2^ heterogeneity test indicated moderate or low heterogeneity (*I*^2^ < 50%); conversely, for substantial heterogeneity (*I*^2^ ≥ 50%), the random effect model was utilized. The resultant composite findings were represented through forest plots. We conducted subgroup analyses to explore the sources of heterogeneity. To verify the reliability of our findings, we performed sensitivity analyses on mental disorders featured in over 10 studies to assess the impact of individual studies on the overall results. To address publication bias, we constructed funnel plots and applied Egger’s test to mental disorder categories with at least 10 estimates (16).

## Results

### Search results

**Figure 1:**
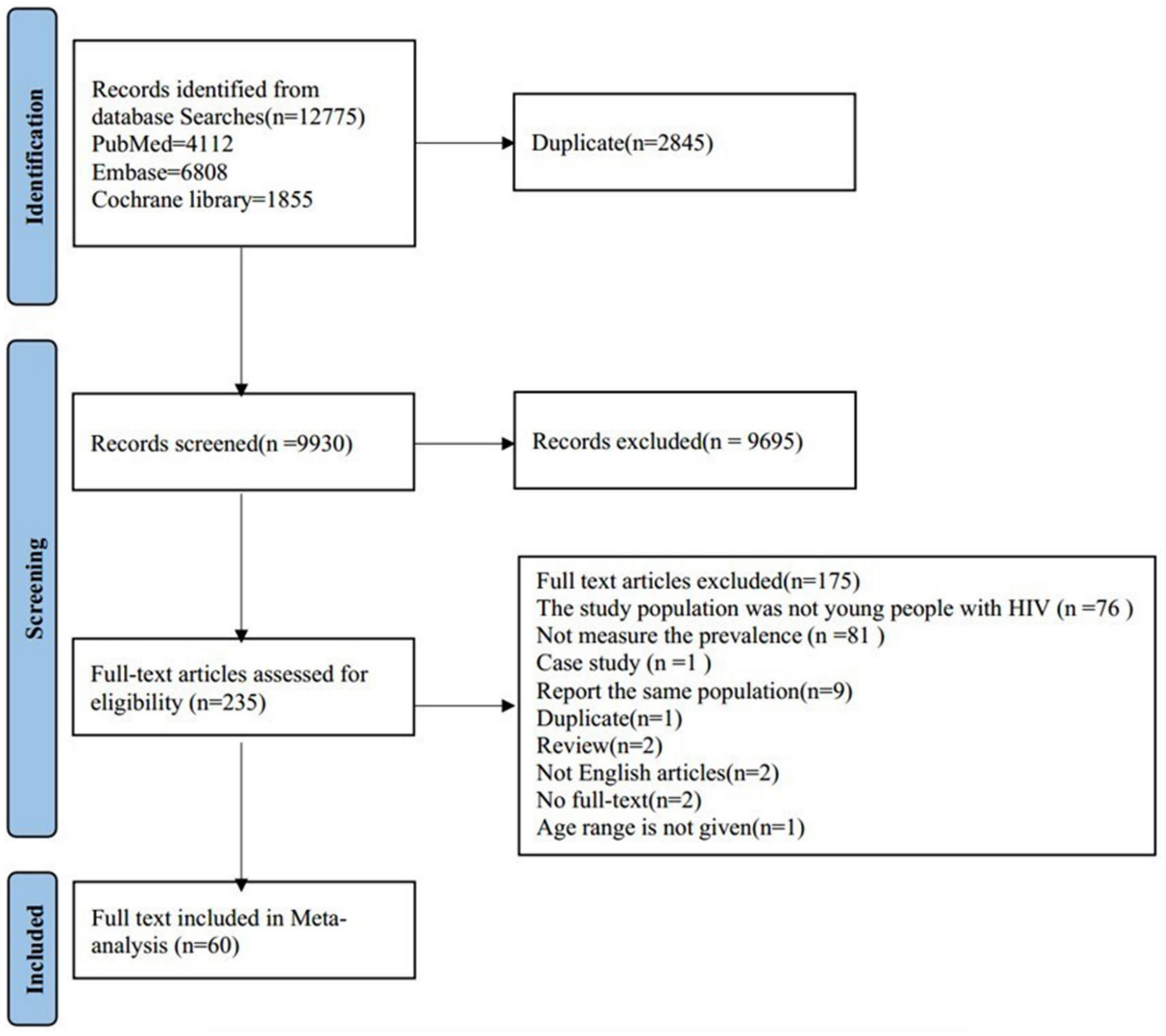
Flowchart of study selection.

We conducted a search across PubMed (4,112 articles), Embase (6,808 articles), and the Cochrane Library (1,855 articles), totaling 12,775 articles. After excluding 2,845 duplicate articles, we reviewed the titles and abstracts of the remaining 9,930 articles, of which 235 articles underwent full-text eligibility screening. Among these, 175 articles were excluded, and ultimately, 60 articles were included in our meta-analysis. The selection process and reasons for exclusion were illustrated in Figure 1.

### Study characteristics

The final 60 studies included in this systematic review and meta-analysis were published in years ranging from 2014 to 2023, with the highest number of studies published in 2020, with 10 studies. The included studies were from 26 countries, of which 15 were from African countries, 7 from Asian countries, 2 from European countries, 1 from North American countries, and 1 from South American countries. The number of studies conducted in Africa, Asia, North America, Europe, and South America was 44, 7, 6, 2, and 1, respectively. Among the 60 studies included, 54 adopted a cross-sectional design, with the remaining comprising 1 case–control and 5 longitudinal studies. Among the 44 studies reporting ART status, 32 had populations on ART, 11 had majorities on ART with over 85% uptake, and only 1 had a population not on ART. Of the 46 studies that reported the survey time, the period of investigation ranged from June 2005 to March 2022 (see Supplementary material 1 Table S1). Among the 60 observational studies, 13 were of high quality, 47 were rated as medium quality, and none were classified as low quality (see Supplementary material 1 Table S2).

### Depression

In this meta-analysis, a total of 51 studies reported on the prevalence of depression in YLWH, as outlined in Table 1. These studies encompassed a collective sample size of 18,535 participants, revealing a pooled prevalence of depression at 24.6% (95% CI: 21.1–28.2%). Notably, there was substantial heterogeneity observed among the studies (*I*^2^ = 98.03%, *p* < 0.001). The majority of these studies were conducted in Africa, accounting for 39 of the total, while South America contributed the least, with only one study. The measurement tools for depression were diverse, with the Patient Health Questionnaire-9 (PHQ-9) being used in 18 studies, the Beck Depression Inventory (BDI) and Child Depression Inventory (CDI) in 6 studies, and Patient Health Questionnaire for Adolescents (PHQ-A), Center for Epidemiologic Studies Depression Scale (CES-D), and Mini International Neuropsychiatric Interview for Children and Adolescents (MINI-KID) in 4 studies. Furthermore, 7 studies utilized different measurement tools, and 3 studies employed two tools concurrently.

**Table 1:** Prevalence estimates for depression among YLWH according to the measurement tool used.

Author, year	Country	Sample size (*n*)	Assessment tool used	Cut-off score	Information on local tool validation	Prevalence estimates
Sohn et al. (17)	Thailand, Vietnam, Malaysia	82	PHQ-9	≥9	NR	23.2%
Olashore et al. (18)	Botswana	622	MINI-KID	NR	NR	23.6%
Nguyen et al. (19)	Mozambique	213	PHQ-A	≥10	NR	11.7%
Ndongo et al. (20)	Cameroon	302	CDI	≥20	NR	26.5%
Mugo et al. (21)	Kenya	938	PHQ-9	≥5	Cronbach’α = 0.70	20.7%
Gamassa et al. (22)	Tanzania	170	PHQ-A	≥5	NR	15.9%
Brooks et al. (23)	Botswana	1,049	PHQ-9	≥5	Cronbach’α = 0.74	42.4%
Arnold et al. (24)	America	163	PHQ-9	≥10	NR	21.5%
Di Gennaro et al. (25)	Mozambique	1,096	PHQ-9	≥11	NR	7.1%
Chory et al. (10)	Kenya	30	PHQ-9	NR	NR	3.3%
Chantaratin et al. (26)	Thailand	100	PHQ-A	≥10	Sensitivity = 0.76 Specificity = 0.81	15.0%
Nyongesa et al. (27)	Kenya	406	PHQ-9	≥10	Cronbach’α = 0.83	28.8%
Mugo et al. (28)	Kenya	96	PHQ-9	≥5	Cronbach’α = 0.88	47.9%
Kohn et al. (29)	America	181	BDI	≥14	NR	39.8%
Girma et al. (30)	Ethiopia	325	PHQ-9	≥10	Cronbach’α = 0.89 Sensitivity = 0.88 Specificity = 0.88	30.2%
Getaye et al. (31)	Ethiopia	431	BDI	≥14	Cronbach’α = 0.85	26.2%
Filiatreau et al. (32)	South Africa	359	CES-D	≥16	Cronbach’α = 0.76	28.1%
Aurpibul et al. (33)	Thailand, Cambodia	193	CDI CES-D	>15 >22	NR NR	20.7%
Ashaba et al. (34)	Uganda	224	MINI-KID	NR	NR	16.5%
Adeyemo et al. (35)	Nigeria	201	MINI-KID	NR	NR	16.9%
Buckley et al. (36)	South Africa	81	PHQ-A	NR	NR	13.6%
Dyer et al. (37)	Kenya	479	PHQ-9	≥5	NR	10.0%
Ekat et al. (38)	Republic of Congo	135	PHQ-9	≥9	NR	38.5%
Haas et al. (8)	South Africa	1,088	PHQ-9	≥10	NR	4.4%
Cavazos-Rehg et al. (39)	Uganda	675	CDI	≥3	NR	52.3%
Yarhere et al. (40)	Nigeria	58	BDI	≥11	NR	44.8%
Agyemang et al. (41)	Ghana	139	PHQ-9	≥5	NR	26.6%
Kinyanda et al. (42)	Uganda	479	YI-4R CASI-5	NR NR	Cronbach’α = 0.88 Cronbach’α = 0.77	5.2%
Kemigisha et al. (43)	Uganda	336	CES-D	≥15	Cronbach’α = 0.85	45.8%
Abebe et al. (44)	Ethiopia	507	BDI	≥21	NR	35.5%
Casale et al. (45)	South Africa	1,053	CDI	NR	Cronbach’α = 0.64	46.0%
Zhou et al. (46)	China	145	CDI	≥19	Cronbach’α = 0.82	32.4%
Wonde et al. (47)	Ethiopia	413	PHQ-9	≥10	NR	31.7%
Coetzee et al. (48)	South Africa	134	RCADS	≥65	Cronbach’α = 0.86	9.7%
Gaitho et al. (11)	Kenya	270	PHQ-9	≥1	NR	52.6%
Ramos et al. (49)	Tanzania	280	PHQ-9	≥10	NR	20.4%
Okawa et al. (50)	Zambia	190	CES-D	≥10	Cronbach’α = 0.74	25.3%
West et al. (51)	South Africa	112	CDI	≥7	NR	10.7%
Earnshaw et al. (52)	South Africa	250	BDI	≥20	Cronbach’α = 0.90	33.8%
Prevost et al. (53)	England	283	HADS	≥8	NR	16.0%
Chenneville et al. (54)	America	131	PHQ-9 PHQ-A	≥10 ≥10	NR NR	21.4%
Woollett et al. (7)	South Africa	343	CDI	≥10	Cronbach’α >0.70	14.3%
Bankole et al. (55)	Nigeria	31	MINI-KID	NR	NR	41.9%
Dow et al. (56)	Tanzania	182	PHQ-9	≥10	NR	12.1%
Fawzi et al. (57)	Rwanda	193	CES-D	≥30	NR	26.4%
Funck-Brentano et al. (58)	France	54	Psychological Interview	NA	NA	16.7%
Côté et al. (59)	Brazil	268	BDI	≥14	Cronbach’α = 0.89	23.9%
Vreeman et al. (60)	Kenya	285	PHQ-9	≥5	NR	18.9%
Lewis et al. (61)	America	166	BDI	≥10	Cronbach’α = 0.87	34.3%
Brown et al. (62)	America	2032	BSI	≥63	Cronbach’α = 0.98	20.6%
Kim et al. (63)	Malawi	562	CDRS-R	≥55	NR	18.9%

NR, Not Reported; NA, Not Applicable; MINI-KID, Mini International Neuropsychiatric Interview for Children and Adolescents; CDI, Children Depression Inventory; BDI, Beck Depression Inventory; PHQ-9, Patient Health Questionnaire-9-item depression instrument; PHQ-A, Patient Health Questionnaire for Adolescents; CES-D, Center for Epidemiologic Studies Depression Scale; YI-4R, Youth Inventory-4R; CASI-5, Child and Adolescent Symptom Inventory-5; RCADS, Revised Children’s Anxiety and Depression Scale; BSI, Brief Symptom Inventory; CDRS-R, Children’s Depression Rating Scale-Revised; HADS, Hospital Anxiety and Depression Scale.

The subgroup analysis of depression, as presented in Table 2, revealed variations in depression prevalence attributed to the measurement tools employed, yielding statistically significant differences among the groups (*p* < 0.001). Studies utilizing MINI-KID, PHQ-A, and BDI demonstrated reduced heterogeneity, particularly among those using PHQ-A (*I*^2^ = 0%, *p* = 0.67). Intriguingly, depression prevalence as determined by the PHQ-9 with a cut-off score of 5 was significantly higher (27.8, 95% CI: 18.0 to 37.5%) compared to a cut-off score of 10 (19.7, 95% CI: 11.3 to 28.1%). Similarly, the depression prevalence assessed by the PHQ-9 (cut-off score of 5) closely mirrored that assessed by the BDI (cut-off score of 14).

**Table 2:** Overall and subgroup analysis of prevalence of mental disorders in YLWH.

Subgroups	Studies, *n*	Sample size, *n*	Prevalence (%)	95%CI (%)	*I*^2^ (%)	*p*-value	Heterogeneity between groups (*p*-value)
Depression							
Overall	51	18,535	24.6	21.1 ~ 28.2	97.99	<0.001	
Continent							0.010
Africa	39	14,737	25.0	20.6 ~ 29.4	98.41	<0.001	
Asia	4	520	22.7	15.5 ~ 29.8	73.36	0.010	
North America	5	2,673	27.2	19.5 ~ 35.0	89.32	<0.001	
Europe	2	337	16.1	12.2 ~ 20.0	0.00	0.900	
South America	1	268	23.9	18.8 ~ 29.0	–	–	
Measurement tools							<0.001
PHQ-9	18	7,456	24.2	17.6 ~ 30.9	98.49	<0.001	
PHQ-A	4	564	13.6	10.8 ~ 16.4	0.00	0.670	
CES-D	4	1,078	31.5	22.0 ~ 41.0	91.69	<0.001	
MINI-KID	4	1,078	21.7	14.5 ~ 28.9	79.19	<0.001	
BDI	6	1861	33.1	28.1 ~ 38.0	73.18	0.002	
CDI	6	2,630	30.4	17.0 ~ 43.8	98.50	<0.001	
CDI&CES-D	1	193	20.7	15.0 ~ 26.4	–	–	
YI-4R&CASI-5	1	479	5.2	3.2 ~ 7.2	–	–	
RCADS	1	134	9.7	4.7 ~ 14.7	–	–	
HADS	1	283	16.0	11.7 ~ 20.3	–	–	
PHQ-9&PHQ-A	1	131	21.4	14.4 ~ 28.4	–	–	
BSI	1	2032	20.6	18.8 ~ 22.4	–	–	
CDRS-R	1	562	18.9	15.7 ~ 22.1	–	–	
Measurement and cut-off score							<0.001
PHQ-9 ≥ 5	7	3,311	27.8	18.0 ~ 37.5	97.92	<0.001	
PHQ-9 ≥ 9	2	217	31.0	16.0 ~ 46.0	83.23	0.010	
PHQ-9 ≥ 10	6	2,532	19.7	11.3 ~ 28.1	98.15	<0.001	
PHQ-9 ≥ 1	1	270	52.6	46.6 ~ 58.6	–	–	
PHQ-9 ≥ 11	1	1,096	7.1	5.6 ~ 8.6	–	–	
PHQ-A ≥ 5	1	170	15.9	10.4 ~ 21.4	–	–	
PHQ-A ≥ 10	2	313	12.6	8.9 ~ 16.3	0.00	0.620	
BDI ≥ 10	1	166	34.3	27.1 ~ 41.5	–	–	
BDI ≥ 11	1	58	44.8	32.0 ~ 57.6	–	–	
BDI ≥ 14	3	880	29.6	20.3 ~ 39.0	85.39	0.001	
BDI ≥ 20	1	250	33.8	27.9 ~ 39.7	–	–	
BDI ≥ 21	1	507	35.5	31.3 ~ 39.7	–	–	
CDI ≥ 3	1	675	52.3	48.5 ~ 56.1	–	–	
CDI ≥ 7	1	112	10.7	5.0 ~ 16.4	–	–	
CDI ≥ 10	1	343	14.3	10.6 ~ 18.0	–	–	
CDI ≥ 19	1	145	32.4	24.8 ~ 40.0	–	–	
CDI ≥ 20	1	302	26.5	21.5 ~ 31.5	–	–	
CES-D ≥ 10	1	190	25.3	19.1 ~ 31.5	–	–	
CES-D ≥ 15	1	336	45.8	40.5 ~ 51.1	–	–	
CES-D ≥ 16	1	359	28.1	23.5 ~ 32.7	–	–	
CES-D ≥ 30	1	193	26.4	20.2 ~ 32.6	–	–	
RCADS≥65	1	134	9.7	4.7 ~ 14.7	–	–	
HADS≥8	1	283	16.0	11.7 ~ 20.3	–	–	
BSI ≥ 63	1	2032	20.6	18.8 ~ 22.4	–	–	
CDRS-R ≥ 55	1	562	18.9	15.7 ~ 22.1	–	–	
Sex							0.420
Male	13	3,181	29.2	20.9 ~ 37.5	95.82	<0.001	
Female	13	2,712	33.7	26.5 ~ 40.8	94.78	<0.001	
Age group							0.440
10 ~ 14	7	1,074	24.7	16.0 ~ 33.5	94.27	<0.001	
15 ~ 19	9	1,647	31.9	17.9 ~ 45.8	97.79	<0.001	
20 ~ 24	3	520	36.2	18.3 ~ 54.1	94.56	<0.001	
Anxiety							
Overall	19	8,585	17.0	11.4 ~ 22.6	97.95	<0.001	
Continent							0.680
Africa	13	5,955	14.0	8.6 ~ 19.4	98.22	<0.001	
Asia	2	130	32.6	0.0 ~ 78.3	95.83	<0.001	
Europe	2	337	24.8	0.0 ~ 54.9	97.44	<0.001	
North America	2	2,163	18.8	7.4 ~ 30.2	89.07	0.002	
Measurement tools							<0.001
GAD-7	6	3,983	16.7	7.9 ~ 25.4	99.00	<0.001	
RCMAS	2	455	15.4	0.0 ~ 33.2	96.78	<0.001	
MASC	1	302	29.1	24.0 ~ 34.2	–	–	
Hopkins questionnaires	1	30	3.3	0.0 ~ 9.7	–	–	
SCAS	1	30	56.7	39.0 ~ 74.4	–	–	
SCARED	1	100	10.0	4.1 ~ 15.9	–	–	
PHQ-A	1	81	3.7	0.0 ~ 7.8	–	–	
YI-4R&CASI-5	1	479	14.7	11.5 ~ 17.9	–	–	
RCADS	1	134	6.7	2.5 ~ 10.9	–	–	
HADS	1	283	40.0	34.3 ~ 45.7	–	–	
BSI	1	2032	13.5	12.0 ~ 15.0	–	–	
MINI-KID	1	622	18.0	15.0 ~ 21.0	–	–	
Suicidal ideation							
Overall	16	5,476	16.8	11.3 ~ 22.4	95.04	<0.001	
Measurement tools							<0.001
PHQ-9	3	397	12.7	2.7 ~ 22.9	86.00	<0.001	
CDI	2	413	29.3	14.8 ~ 43.8	89.54	0.002	
PHQ-A	2	251	35.8	25.3 ~ 46.3	63.39	0.010	
MINI-KID	3	1,597	15.3	6.3 ~ 24.3	95.51	<0.001	
CES-D	1	336	7.7	4.8 ~ 10.6	–	–	
CIDI	1	413	5.7	3.5 ~ 7.9	–	–	
YSR	1	218	21.1	15.7 ~ 26.5	–	–	
CDRS-R	1	562	7.1	5.0 ~ 9.2	–	–	
Sample size							0.260
<300	9	1,371	19.8	12.7 ~ 26.8	93.22	<0.001	
≥300	7	4,105	13.3	4.7 ~ 21.9	96.36	<0.001	
Suicidal attempts							
Overall	9	3,603	9.7	4.0 ~ 15.4	96.09	<0.001	
Sample size							0.210
<300	4	780	14.1	3.9 ~ 24.3	97.10	<0.001	
≥300	5	2,823	6.5	0.6 ~ 12.4	96.04	<0.001	
Measurement tools							<0.001
MINI-KID	5	2,611	6.0	0.0 ~ 12.3	96.57	<0.001	
CIDI	1	413	3.4	1.7 ~ 5.1	–	–	
YSR	1	218	21.1	15.7 ~ 26.5	–	–	
Lifetime suicidal ideation							
Overall	2	614	29.7	23.7 ~ 35.7	58.81	0.120	
Lifetime suicidal attempts							
Overall	5	1,344	12.9	2.8 ~ 23.1	96.90	<0.001	
Measurement tools							<0.001
MINI-KID	2	593	2.2	0.9 ~ 3.4	15.42	0.280	
PHQ-A	1	170	14.7	9.4 ~ 20.0	–	–	
CIDI	1	413	16.9	13.3 ~ 20.5	–	–	
Sample size							
<300	3	539	15.3	0.0 ~ 31.6	97.48	<0.001	
≥300	2	805	9.8	0.0 ~ 23.6	97.94	<0.001	
PTSD							
Overall	8	3,313	10.5	5.8 ~ 15.2	94.77	<0.001	
Measurement tools							0.890
PC-PTSD	3	2,315	11.8	3.0 ~ 20.6	97.99	<0.001	
CPC	3	536	9.1	0.0 ~ 20.5	87.55	<0.001	
UCLA PTSD-RI	2	462	12.0	9.0 ~ 14.9	0.00	0.360	
Sample size							0.300
<300	5	786	12.7	6.2 ~ 19.1	89.29	<0.001	
≥300	3	2,527	7.6	0.7 ~ 14.6	97.71	<0.001	
ADHD							
Overall	4	957	5.0	3.1 ~ 7.0	42.34	0.160	

The prevalence of depression also demonstrated significant geographic variation. The highest pooled prevalence was observed in North America (27.2, 95% CI: 19.5–35.0%), and the lowest in Europe (16.1, 95% CI: 12.2–20.0%). Notably, heterogeneity was considerably reduced in studies from Asia, North America, and Europe, with respective *I*^2^ values of 73.36, 89.32, and 0%. Gender and age-based Subgroup analyses showed that the prevalence of depression in females (33.7, 95% CI: 26.5–40.8%) was higher than in males (29.2, 95% CI: 20.9–37.5%), but the difference did not reach statistical significance (*p* = 0.42). Additionally, higher prevalence rates were observed in older youth, with the 20–24 age group showing a prevalence of 36.2% (95% CI: 18.3–54.1%) and the 15–19 age group (31.9, 95% CI: 17.9–45.8%), compared to the 10–14 age group (24.7, 95% CI: 16.0–33.5%); However, these differences did not achieve statistical significance either (*p* = 0.44).

### Anxiety

The meta-analytic synthesis of anxiety prevalence, conducted across 19 studies as delineated in Table 3, revealed a summary prevalence of 17.0% (95% CI: 11.4–22.6%) from a collective sample of 8,585 participants. This analysis uncovered considerable heterogeneity across studies (*I*^2^ = 97.95%, *p* < 0.001). Samples were sourced from a variety of geographic regions: Asia (*n* = 2), Africa (*n* = 13), North America (*n* = 2), and Europe (*n* = 2). Six studies predominantly utilized the Generalized Anxiety Disorder-7 (GAD-7) scale with a threshold of 10, whereas the Revised Children’s Manifest Anxiety Scale (RCMAS) was used in 2 studies. Notably, 1 study did not specify the measurement tool employed, another utilized two different tools concurrently, and the remaining 10 studies incorporated various other instruments.

**Table 3:** Prevalence estimates for anxiety among YLWH according to the measurement tool used.

Author, year	Country	Sample size (*n*)	Assessment tool used	Cut-off score	Information on local tool validation	Prevalence estimates
Olashore et al. (64)	Botswana	622	MINI-KID	NR	NR	18.0%
Nguyen et al. (19)	Mozambique	213	GAD-7	≥10	NR	12.2%
Ndongo et al. (20)	Cameroon	302	MASC	NR	NR	29.1%
Brooks et al. (23)	Botswana	1,049	GAD-7	≥5	Cronbach’α = 0.81	32.1%
Chory et al. (10)	Kenya	30	Hopkins questionnaires	NR	NR	3.3%
Satriawibawa et al. (9)	Indonesia	30	SCAS	≥60	NR	56.7%
Di Gennaro et al. (25)	Mozambique	1,096	GAD-7	≥10	NR	10.3%
Chantaratin et al. (26)	Thailand	100	SCARED	≥25	Sensitivity = 0.79 Specificity = 0.82	10.0%
Nyongesa et al. (27)	Kenya	406	GAD-7	≥10	Cronbach’α = 0.86	19.0%
Buckley et al. (36)	South Africa	81	PHQ-A	NR	NR	3.7%
Haas et al. (8)	South Africa	1,088	GAD-7	≥10	NR	2.2%
Kinyanda et al. (42)	Uganda	479	YI-4R CASI-5	NR	Cronbach’α = 0.88 Cronbach’α = 0.77	14.7%
Coetzee et al. (48)	South Africa	134	RCADS	≥65	Cronbach’α = 0.89	6.7%
West et al. (51)	South Africa	112	RCMAS	≥10	NR	6.3%
Prevost et al. (53)	England	283	HADS	≥8	NR	40.0%
Chenneville et al. (54)	America	131	GAD-7	≥10	NR	25.2%
Woollett et al. (7)	South Africa	343	RCMAS	NR	NR	24.5%
Funck-Brentano et al. (58)	France	54	NR	NR	NR	9.3%
Brown et al. (62)	America	2032	BSI	≥63	Cronbach’α = 0.98	13.5%

NR, Not Reported; MINI-KID, Mini International Neuropsychiatric Interview for Children and Adolescents; GAD-7, Generalized Anxiety Scale-7; MASC, Multidimensional Anxiety Scale for Children; SCAS, Spence children’s anxiety scale; SCARED, Screen for Child Anxiety Related Disorder; PHQ-A, Patient Health Questionnaire for Adolescents; YI-4R, Youth Inventory-4R; CASI-5, Child and Adolescent Symptom Inventory-5; RCADS, Revised Children’s Anxiety and Depression Scale; RCMAS, Revised Children’s Manifest Anxiety Scale; HADS, Hospital Anxiety and Depression Scale; BSI, Brief Symptom Inventor.

In the stratified meta-analyses by continent and assessment instruments (Table 2), we explored the factors contributing to the observed heterogeneity and detailed the anxiety prevalence across continents. The pooled prevalence of anxiety in YLWH varied considerably, ranging from 14.0% in Africa (95% CI: 8.6–19.4%) to 32.6% in Asia (95% CI: 0.0–78.3%); however, these intercontinental differences did not reach statistical significance (*p* = 0.68). Subgroup analyses further revealed variations in anxiety prevalence depending on the measurement tools used, with significant differences detected (*p* < 0.001). The overall pooled prevalence and the prevalence determined using the GAD-7 (16.7, 95% CI: 7.9–25.4%) exhibited similarity.

### Suicidality

A total of 5,476 participants from 16 studies assessed suicidal ideation, while 614 participants from 2 studies evaluated lifetime suicidal ideation, as noted in Table 4. Regarding suicide attempts, 9 studies (totaling 3,603 participants) reported on suicide attempt prevalence, and 5 studies (with 1,344 participants) on lifetime suicide attempt prevalence (Table 4). As shown in Table 2, random effect prevalence estimates varied from 9.7% (95% CI: 4.0–15.4%) for lifetime attempts to 29.7% (95% CI: 23.7–35.7%) for suicidal ideation prevalence. Heterogeneity measures were notably high across all outcomes, with *I*^2^ ranging from 58.81 to 96.90%.

**Table 4:** Prevalence estimates for suicidality among YLWH according to the measurement tool used.

Author, year	Country	Sample size (*n*)	Assessment tool used	Cut-off score	Information on local tool validation	Prevalence estimates
Sohn et al. (17)	Thailand, Vietnam, Malaysia	82	PHQ-9	NR	NR	SI 17.1% LTSA 14.7%
Ndongo et al. (20)	Cameroon	302	CDI	NR	NR	SI 36.4%
Arnold et al. (24)	America	168	NR	NR	NR	SA 23.5% LTSA 30.4%
Gamassa et al. (22)	Tanzania	170	PHQ-A	NR	NR	SI 31.2%
Gennaro et al. (25)	Mozambique	1,096	Self-report	NA	NA	SI 5.9%
Olashore et al. (64)	Botswana	622	MINI-KID	NR	NR	SA 18.8%
Namuli et al. (65)	Uganda	111	CDI	The score of item 9 = 1 or 2	NR	SI 21.6%
Adeyemo et al. (35)	Nigeria	201	MINI-KID	NR	NR	SI 14.9% LTSI 33.3% SA 1.0% LTSA 1.5%
Buckley et al. (36)	South Africa	81	PHQ-A	NR	NR	SI 42.0%
Rukundo et al. (66)	Uganda	392	MINI-KID	NR	NR	SA 1.8% LTSA 2.8%
Kemigisha et al. (43)	Uganda	336	CES-D	NR	Cronbach’α = 0.85	SI 7.7%
Casale et al. (45)	South Africa	1,053	MINI-KID	NR	Cronbach’α = 0.88	SI 8.0% SA 4.0%
Wonde et al. (47)	Ethiopia	413	CIDI	NR	NR	SI 5.7% LTSI 27.1% SA 3.4% LTSA 16.9%
López et al. (67)	America	45	PHQ-9	NR	NR	SI 20.0%
Gaitho et al. (11)	Kenya	270	PHQ-9	NR	NR	SI 4.4%
Woollett et al. (7)	South Africa	343	MINI-KID	NR	Cronbach’α = 0.75	SI 24.0% SA 5.0%
Fawzi et al. (57)	Rwanda	193	Self-report	NA	NA	SI 10.9% SA 11.9%
Ng et al. (68)	Rwanda	218	YSR	NR	Cronbach’α = 0.89	SI 21.1% SA 21.1%
Kim et al. (63)	Malawi	562	CDRS-R	The score of question 13 > 2	NR	SI 7.1%

NR, Not Reported; NA, Not Applicable; SI, Suicidal ideation; LTSI, Lifetime suicidal ideation; SA, Suicidal attempts, LTSA, Lifetime suicidal attempts; PHQ-9, Patient Health Questionnaire-9-item depression instrument; CES-D, Center for Epidemiologic Studies Depression Scale; PHQ-A, Patient Health Questionnaire for Adolescents; CIDI, World Health Organization Composite International Diagnostic Interview; CDI, Children Depression Inventory; CDRS-R, Children’s Depression Rating Scale-Revised; YSR, Youth Self-Report Internalizing Subscale; MINI-KID, Mini International Neuropsychiatric Interview for Children and Adolescents.

Subgroup analysis revealed significant differences in suicidal ideation prevalence when measured with the PHQ-A (35.8, 95%CI: 25.3–46.3%) and CDI (29.3, 95%CI: 14.8–43.8%), compared to the MINI-KID (15.3, 95%CI: 6.3–24.3%) and PHQ-9 (12.7, 95%CI: 2.7–22.8%). Additionally, marked differences were noted in the prevalence of suicidal and lifetime suicide attempts across measurement tools (*p* < 0.001). Analysis stratified by sample size revealed that studies with smaller sample sizes (<300) indicated higher rates of suicidal ideation, attempts, and lifetime attempts compared to those with larger sample sizes (≥300).

### PTSD and ADHD

Eight studies reported on the prevalence of PTSD, as indicated in Table 5, encompassing a combined sample size of 3,313 participants. There was a notable variation in the reported prevalence of PTSD, with substantial heterogeneity observed (*I*^2^ = 94.77%, *p* < 0.001). Consequently, a random effects model was employed to aggregate the prevalence rates, yielding an overall prevalence of 10.5% (95% CI: 5.8–15.2%). Across the included studies, a total of three different measurement tools were utilized, with three studies employing both the Primary care-PTSD screen (PC-PTSD) and Child Post-Traumatic Stress Disorder Checklist (CPC), while the remaining two studies utilized the University of California Los Angeles PTSD Exposure questionnaire (UCLA PTSD-RI) for assessment.

**Table 5:** Prevalence estimates for PTSD among YLWH according to the measurement tool used.

Author, year	Country	Sample size (*n*)	Assessment tool used	Cut-off score	Information on local tool validation	Prevalence estimates
Gennaro et al. (25)	Mozambique	1,096	PC-PTSD	≥3	NR	14.70%
Buckley et al. (36)	South Africa	81	CPC	≥20	NR	22.20%
Haas et al. (8)	South Africa	1,088	PC-PTSD	≥3	NR	3.50%
Ramos et al. (49)	Tanzania	280	UCLA PTSD-RI	≥18	NR	13.20%
West et al. (51)	South Africa	112	CPC	≥3	NR	2.70%
Chenneville et al. (54)	America	131	PC-PTSD	≥3	NR	18.30%
Woollett et al. (7)	South Africa	343	CPC	NR	Cronbach’α = 0.89	4.70%
Dow et al. (56)	Tanzania	182	UCLA PTSD-RI	≥18	Cronbach’α ≥ 0.90	10.40%

NR, Not Reported; PTSD, Post-traumatic stress disorder; CPC, Child Post-Traumatic Stress Disorder Checklist; PC-PTSD, Primary care-PTSD screen; UCLA PTSD-RI, University of California Los Angeles PTSD Exposure questionnaire.

Table 6 displays the ADHD prevalence rates from all studies included. Heterogeneity testing yielded low to moderate levels (*I*^2^ = 42.34%, *p* = 0.16), necessitating the use of a fixed effects model for the analysis. ADHD prevalence in YLWH was determined to be 5.0% (95% CI: 3.1–7.0%).

**Table 6:** Prevalence estimates for ADHD among YLWH according to the measurement tool used.

Author, year	Country	Sample size (*n*)	Assessment tool used	Cut-off score	Information on local tool validation	Prevalence estimates
Zhou et al. (69)	China	479	SDQ	NR	Cronbach’α = 0.72	6.2%
Kinyanda et al. (42)	Uganda	325	YI-4R CASI-5	NR	Cronbach’α = 0.88 Cronbach’α = 0.77	6.4%
Gentz et al. (70)	Namibia	99	SDQ	NR	NR	4.0%
Funck-Brentano et al. (58)	France	54	Psychological interview	NA	NA	1.9%

NR, Not reported; NA, Not Applicable; YI-4R, Youth Inventory-4R; CASI-5, Child and Adolescent Symptom Inventory-5; SDQ, The Strengths and Difficulties Questionnaire.

Additionally, subgroup analysis presented in Table 2, which focused on the measurement tools for PTSD, showed that the prevalence rates assessed by different tools were comparable, with no statistically significant variations found across groups (*p* = 0.22). It was noted that studies with smaller sample sizes (<300) reported higher prevalence rates compared to those with larger sample sizes (≥300), though these differences were not statistically significant (*p* = 0.30).

### Sensitivity analysis and publication bias

The sensitivity analysis revealed that no individual study significantly influenced the aggregated prevalence rates of depression, anxiety, or suicidal ideation, indicating a stable overall outcome (see Supplementary material 2 Figures S3, S4, S6). Funnel plots for depression, anxiety and suicidal ideation showed significant asymmetry (see Supplementary material 2 Figures S12–S14). Egger’s test indicated a significant risk of publication bias in the pooled prevalence rates of depression, anxiety, and suicidal ideation (*p* < 0.05).

## Discussion

This systematic review and meta-analysis included a total of 60 studies from 26 different countries, focusing on the mental disorders among YLWH. In general, the prevalence of depression, anxiety, suicidal ideation, lifetime suicidal ideation, suicidal attempts, lifetime suicidal attempts, PTSD, and ADHD was assessed. To our knowledge, this is the first systematic evaluation of mental disorders in YLWH globally.

The meta-analysis revealed that depression and anxiety are the predominant mental disorders among YLWH, with the pooled prevalence rates being 24.6% (95% CI: 21.1–28.2%) for depression and 17.0% (95% CI: 11.4–22.6%) for anxiety, respectively (71, 72). Compared to general young people, depression and anxiety are 2.34 and 2.62 times more prevalent in YLWH in the current study, respectively. Considering the large number of YLWH worldwide, depression and anxiety issues should be the major health priorities for this population. The greater prevalence of depression in YLWH could be attributed to several factors. The first is the release of monoamines and increased levels of cytokines that promote inflammation (73). Additionally, depression’s start is significantly linked to psychosocial stigma-related factors (74). Similarly, there is proof that stigma connected to HIV and anxiety are strongly correlated (75). Consistent with the reported rates of depression in the general young population, our meta-analysis found that depression is more prevalent among female YLWH than their male counterparts, and higher among older youth compared to their younger peers (76, 77). The gender disparity in depression prevalence may stem from differing societal expectations and moral standards for men and women, alongside varying degrees of social discrimination and psychological stress. Our study found that depression prevalence varied from 16.1 to 27.2%, while anxiety prevalence ranged from 14.0 to 32.6% across different continents. Instead of being only due to changes in geography, these disparities could also be ascribed to variations in social level, religious affiliation, and cultural diversity. The included studies use a variety of measurement tools to measure depression and anxiety, and most of these measurement tools had unclear sensitivity and accuracy. We therefore conducted subgroup analyses of depression and anxiety based on measurement tools. Surprisingly, the prevalence of depression and anxiety measured by different measurement tools and even the prevalence of depression measured using the same measurement tools but with different cut-off scores varied considerably. So it is important to use validated assessment methods carefully because doing otherwise can lead to inaccurate or misleading results (78).

While antiretroviral therapy offers considerable benefits, the issue of suicidality among HIV/AIDS patients continues to pose a significant public health challenge, especially in developing countries (79, 80). The meta-analysis showed that 16.8% of young people living with HIV experienced suicidal ideation, and 9.7% attempted suicide, rates significantly exceeding those in the general population (81). However, a recent review on suicidal ideation in Chinese adults with HIV found the incidence to be 30.6% (82). This may be because in many countries, adults and middle-aged individuals, who bear significant economic and family responsibilities, face high social pressures and are at greater risk of suicide. Our analysis of the available data revealed that 29.7% of young people living with HIV (YLWH) reported experiencing lifetime suicidal ideation, while 12.9% reported lifetime suicide attempts. Previous research indicates that both physiological factors (HIV infection status, low CD4 cell counts, opportunistic infections, adverse reactions to antiretroviral therapy) and psychosocial factors (depression, high stress, low social support, violence, discrimination exposure) significantly contribute to suicidality risk in individuals with HIV (83–85). Among YLWH, studies have shown a higher prevalence of opportunistic infections and a greater risk of stigma and discrimination (74, 86). Therefore, suicidality among YLWH should require urgent attention in terms of prevention and treatment.

This meta-analysis offers, to the best of our knowledge, the first global assessment of PTSD and ADHD among YLWH. Data from 8 studies were combined to evaluate the prevalence of PTSD in YLWH in the current meta-analysis. The prevalence of PTSD from the 2 studies was merged in a meta-analysis examining psychiatric disorders in HIV-infected individuals less than 19 years of age in sub-Saharan Africa (SSA), and the resuls was 3.0% (87), which is substantially lower than what we measured (10.5%). However, a different meta-analysis with adult HIV-infected patients as the research group found that the prevalence of PTSD was 2.40 times greater than ours (88). There is one rationale why this might be the case. According to a study conducted on HIV-infected patients, PTSD is linked to HIV infection duration (89). The fact that young people have had an HIV diagnosis for a shorter amount of time may thus account for this difference. Despite this, YLWH still has a much higher prevalence of PTSD than the general population (3.9%) (90) and other vulnerable groups, such as cancer patients (5.1%) (91). Our meta-analysis revealed that 5.0% of YLWH had ADHD, which was less than what was previously reported in kids and adolescents with HIV in the prior review (92). This may be a result of the beneficial effects of early ART on the developing brain (93), in comparison to the pre-ART era, the introduction of ART both prenatally and postnatally may have considerably reduced the rate of ADHD among YLWH.

Identifying several limitations in this meta-analysis is crucial. Firstly, because there were variations in the research populations, study designs, measurement tools, and their cutoff values, there was a considerable degree of heterogeneity among the included studies. Secondly, the majority of the prevalence of mental disorders found in this study was derived from research conducted in African nations, which may limit the applicability of our findings elsewhere. And more excellent research from other continents is anticipated to be available in the future, which will allow us to update our findings even further.

## Conclusion

This systematic review and meta-analysis found a significantly higher prevalence of mental disorders among YLWH, highlighting the benefits of early screening and intervention, particularly for females. More high-quality longitudinal studies are required to explore the reasons behind the increased prevalence of mental disorders in this group. Additionally, research into improved screening, prevention, and intervention methods for these issues is necessary.

## Data Availability

The original contributions presented in the study are included in the article/Supplementary material, further inquiries can be directed to the corresponding author.
