# Supplementary material for: Prevalence of mental disorders among young people living with HIV: a systematic review and meta-analysis
Source: Front Public Health. 2024 Aug 21;12:1392872. doi: 10.3389/fpubh.2024.1392872 (PMC11372585; doi:10.3389/fpubh.2024.1392872)
Supplement: Supplementary file 1 [file Data_Sheet_1.docx]

Table S1 : Summary of included studies reporting the prevalence of Mental disorders among young people living with HIV

| Author, year | Country | Study design | ART status | Age range(years) | Male(%) | Mean age/Median age(years) | Survey time | Sample size (n) |
| --- | --- | --- | --- | --- | --- | --- | --- | --- |
| Sohn et al.2023(1) | Thailand Vietnam Malaysia | longitudinal | On ART | 16~24 | 40.2 | 22.0 (20.0-23.0) | NR | 82 |
| Olashore et al.2023(2) | Botswana | CS | On ART | 12~19 | 54.3 | 17.7±1.6 | January 2020 to December 2021 | 622 |
| Nguyen et al.2023(3) | Mozambique | CS | On ART | 15~19 | 31.5 | 18.0(16.0-19.0) | June 2019 to December 2019 | 213 |
| Ndongo et al.2023(4) | Cameroon | CS | On ART | 10~19 | 47.4 | 15.2 (12.0-17.5) | December 2021 to March 2022 | 302 |
| Mugo et al.2023(5) | Kenya | longitudinal | On ART | 15~24 | NR | 18.0(16.0-21.0) | 2019 to 2020 | 938 |
| Gamassa et al.2023(6) | Tanzania | CS | NR | 10~19 | 56.5 | NR | February 2021 to April 2021 | 170 |
| Brooks et al.2023(7) | Botswana | CS | NR | 12~20 | NR | NR | January to December 2019 | 1049 |
| Arnold et al.2023(8) | America | CS | 89.1% On ART | 12~24 | NR | NR | May 2017 to May 2020 | 163 |
| Zhou et al.2023(9) | China | CS | On ART | 11~17 | 52.0 | 14.4±1.9 | July 2021 to August 2021 | 325 |
| Olashore et al.2022(10) | Botswana | CS | On ART | 12~19 | 54.3 | 17.7±1.6 | January 2020 to December 2021 | 622 |
| Chory et al.2022(11) | Kenya | longitudinal | On ART | 10~19 | 43.3 | NR | NR | 30 |
| Wayan Eka et al.2022(12) | Indonesia | CS | NR | 13~18 | NR | NR | December 2020 | 30 |
| Gennaro et al.2022(13) | Mozambique | CS | On ART | 10~24 | 36.3 | 18.5(12.0-24.0) | November 2019 to November 2021 | 1096 |
| Chantaratin et al.2022(14) | Thailand | CS | 98.0% On ART | 15~24 | 35.0 | 19.0 (17.0-22.0) | June 2019 to January 2020 | 100 |
| Nyongesa et al.2021(15) | Kenya | CS | On ART | 18~24 | 43.4 | 20.8 ±2.2 | November 2018 to September 2019 | 406 |
| Namuli et al.2021(16) | Uganda | CS | 87.8% On ART | 13~18 | NR | NR | January 2014 to March 2014 | 111 |
| Mugo et al.2021(17) | Kenya | CS | On ART | 14~24 | 22.0 | NR | December 2017 to April 2019 | 96 |
| Kohn et al.2021(18) | America | longitudinal | Not On ART | 18~24 | 80.7 | NR | April 2008 to July 2010 | 181 |
| Girma et al.2021(19) | Ethiopia | CS | On ART | 15~24 | 61.2 | 20.2±2.8 | March 2020 to June 2020 | 325 |
| Getaye et al.2021(20) | Ethiopia | CS | On ART | 15~24 | 40.4 | NR | February 2020 to March 2020 | 431 |
| Filiatreau et al.2021(21) | South Africa | CS | NR | 12~24 | 29.8 | 21.0 (16.0-23.0) | April 2019 to August 2019 | 359 |
| Author, year | Country | Study design | ART status | Age range(years) | Male(%) | Mean age/Median age(years) | Survey time | Sample size (n) |
| Aurpibul et al.2021(22) | Thailand Cambodia | CS | On ART | 10~24 | 39.5 | 15.1 (13.0-17.0) | NR | 193 |
| Ashaba et al.2021(23) | Uganda | CS | On ART | 13~17 | 42.0 | 14.9±1.4 | NR | 224 |
| Adeyemo et al.2020(24) | Nigeria | CS | NR | 10~19 | 61.7 | 13.9±2.5 | January 2016 to July 2016 | 201 |
| Buckley et al.2020(25) | South Africa | CS | On ART | 13~19 | 44.4 | 16.0(15.0-18.0) | October 2016 to March 2017 | 81 |
| Dyer et al.2020(26) | Kenya | CS | NR | 10~24 | 35.2 | NR | NR | 479 |
| Ekat et al.2020(27) | Congo | CS | On ART | 10~19 | 49.6 | NR | April 2018 to July 2018 | 135 |
| Haas et al.2020(28) | South Africa | CS | On ART | 10~19 | 50.5 | 13.0(10.0-15.0) | February 2018 to January 2020 | 1088 |
| Cavazos-Rehg et al.2020(29) | Uganda | CS | On ART | 10~16 | 43.6 | NR | 2012 to 2017 | 675 |
| Yarhere et al.2020(30) | Nigeria | CS | NR | 10~18 | 87.9 | NR | NR | 58 |
| Rukundo et al.2020(31) | Uganda | CS | NR | 12~17 | 46.7 | NR | NR | 392 |
| Pilania R et al.2020(32) | India | CS | On ART | 10~18 | 64.3 | 13.5±2.3 | November 2017 to March 2019 | 101 |
| Agyemang et al.2020(33) | Ghana | CS | On ART | 13~19 | 43.9 | NR | October 2016 to February 2017 | 139 |
| Kinyanda et al.2019(34) | Uganda | CS | 94.6% On ART | 12~17 | 47.0 | NR | NR | 479 |
| Kemigisha et al.2019(35) | Uganda | CS | 95.2% On ART | 10~19 | 37.8 | 13.0(11.0-16.0) | March 2017 to May 2017 | 336 |
| Abebe et al.2019(36) | Ethiopia | CS | On ART | 15~24 | 46.4 | 18.6±3.0 | May 2016 to June 2016 | 507 |
| Casale et al.2019(37) | South Africa | CS | On ART | 10~19 | 45.2 | 13.8 ±2.8 | 2014 to 2015 | 1053 |
| Zhou et al.2019(38) | China | CS | NR | 11~18 | 57.2 | 16.0±1.9 | NR | 145 |
| Wonde et al.2019(39) | Ethiopia | CS | On ART | 15~24 | 36.8 | 20.9±2.9 | May 2018 to June 2018 | 413 |
| Coetzee et al.2019(40) | South Africa | CS | On ART | 11~18 | 41.8 | 14.3±1.9 | NR | 134 |
| Gaitho et al.2018(41) | Kenya | CS | On ART | 10~19 | 53.7 | 14.8±2.6 | August 2016 to December 2016 | 270 |
| Ramos et al.2018(42) | Tanzania | CS | 92.8% On ART | 11~24 | 44.6 | 16.3(14.7-18.6) | December 2013 to May 2014 | 280 |
| Okawa et al.2018(43) | Zambia | CS | 94.2% On ART | 15~19 | 42.1 | NR | April 2014 to July 2014 | 190 |
| Lopez et al.2018(44) | America | CS | NR | 18~24 | NR | NR | 2012 | 45 |
| Author, year | Country | Study design | ART status | Age range(years) | Male(%) | Mean age/Median age(years) | Survey time | Sample size (n) |
| West et al.2018(45) | South Africa | CS | NR | 13~19 | NR | NR | June 2013 to June 2016 | 112 |
| Earnshaw et al.2018(46) | South Africa | CS | NR | 13~24 | 45.6 | NR | November 2015 to July 2016 | 250 |
| Prevost et al.2018(47) | England | CS | 86.0% On ART | 13~21 | 54.0 | 16.0(15.0-18.0) | 2013 to 2015 | 283 |
| Chenneville et al.2018(48) | America | CS | On ART | 13~24 | 55.7 | 20.6±2.7 | July 2016 to March 2017 | 131 |
| Woollett et al.2017(49) | South Africa | CS | 88.0% On ART | 13~19 | 47.2 | 16.0(12.0-19.0) | NR | 343 |
| Gentz et al.2017(50) | Namibia | CS | On ART | 12~18 | 47.5 | 14.3 ±1.8 | July 2013 to March 2014 | 99 |
| Bankole et al.2017(51) | Nigeria | CS | NR | 11~16 | NR | NR | NR | 31 |
| Dow et al.2016(52) | Tanzania | CS | 91.8% On ART | 12~24 | 45.6 | 17.2±2.9 | December 2013 to May 2014 | 182 |
| Fawzi et al.2016(53) | Rwanda | CS | On ART | 10~17 | 50.3 | NR | NR | 193 |
| Funck Brentano et al.2016(54) | France | CS | 93.0% On ART | 14~20 | 42.6 | 16.0(14.0-20.0) | June 2005 to December 2007 | 54 |
| Côté.J et al.2016(55) | Brazil | longitudinal | On ART | 13~21 | 41.0 | 16.0±1.9 | May 2011 to March 2012 | 268 |
| Vreeman et al.2015(56) | Kenya | CS | On ART | 10~14 | 48.4 | 12.3 ±1.5 | March 2013 to September 2013 | 285 |
| Lauren C et al.2015(57) | Rwanda | CC | NR | 10~17 | NR | NR | March 2012 to December 2012 | 218 |
| Lewis et al.2015(58) | America | CS | NR | 13~21 | 47.0 | 16.8 ±2.5 | NR | 166 |
| Brown et al.2015(59) | America | CS | NR | 12~24 | 67.4 | 20.3± 2.1 | December 2009 to January 2012 | 2032 |
| Kim et al.2014(60) | Malawi | CS | On ART | 12~18 | 44.0 | 14.5±2.0 | January 2012 to August 2012 | 562 |

Table S2 Literature quality assessment

| Study | Response | | | | | | | | | | | |
| --- | --- | --- | --- | --- | --- | --- | --- | --- | --- | --- | --- | --- |
|  | Q1 | Q2 | Q3 | Q4 | Q5 | Q6 | Q7 | Q8 | Q9 | Q10 | Q11 | Total |
| Sohn et al.2023 | Y | Y | N | Y | N | N | N | Y | N | Y | U | 6 |
| Olashore et al.2023 | Y | Y | Y | Y | N | N | N | Y | N | Y | N | 7 |
| Nguyen et al.2023 | Y | Y | Y | Y | N | N | N | Y | N | Y | N | 7 |
| Ndongo et al.2023 | Y | N | Y | Y | N | N | N | Y | N | Y | N | 6 |
| Mugo et al.2023 | Y | Y | Y | Y | N | N | N | Y | N | Y | N | 7 |
| Gamassa et al.2023 | Y | Y | Y | Y | N | N | N | Y | N | Y | N | 7 |
| Brooks et al.2023 | Y | N | Y | Y | N | N | N | Y | N | Y | N | 6 |
| Arnold et al.2023 | Y | Y | Y | Y | N | N | N | Y | N | Y | N | 7 |
| Zhou et al.2023 | Y | Y | Y | Y | N | N | N | N | N | Y | N | 6 |
| Olashore et al.2022 | Y | Y | Y | Y | N | N | N | Y | N | Y | N | 7 |
| Chory et al.2022 | Y | Y | N | Y | N | N | N | N | N | Y | N | 5 |
| Wayan Eka et al.2022 | Y | N | Y | Y | N | N | N | Y | N | Y | N | 6 |
| Gennaro et al.2022 | Y | Y | Y | Y | N | N | N | Y | N | Y | N | 7 |
| Chantaratin et al.2022 | Y | N | Y | Y | N | N | N | Y | N | Y | N | 6 |
| Nyongesa et al.2021 | Y | Y | Y | Y | N | N | N | Y | N | Y | N | 7 |
| Namuli et al.2021 | Y | Y | Y | Y | N | N | N | Y | N | Y | N | 7 |
| Mugo et al.2021 | Y | Y | Y | Y | N | N | N | Y | N | Y | N | 7 |
| Kohn et al.2021 | Y | N | Y | Y | N | N | N | Y | N | Y | N | 6 |
| Girma et al.2021 | Y | Y | Y | Y | N | Y | N | Y | N | Y | N | 8 |
| Getaye et al.2021 | Y | Y | Y | Y | N | N | N | Y | N | Y | N | 7 |
| Study | Response | | | | | | | | | | | |
|  | Q1 | Q2 | Q3 | Q4 | Q5 | Q6 | Q7 | Q8 | Q9 | Q10 | Q11 | Total |
| Filiatreau et al.2021 | Y | Y | Y | Y | N | N | N | Y | N | Y | N | 7 |
| Aurpibul et al.2021 | Y | Y | N | Y | N | N | N | Y | N | Y | N | 6 |
| Ashaba et al.2021 | Y | Y | N | Y | N | Y | Y | Y | N | Y | N | 8 |
| Adeyemo et al.2020 | Y | N | Y | Y | N | N | N | Y | N | Y | N | 6 |
| Buckley et al.2020 | Y | Y | Y | Y | N | Y | N | Y | N | Y | N | 8 |
| Dyer et al.2020 | Y | Y | N | Y | N | N | N | N | N | Y | N | 5 |
| Ekat et al.2020 | Y | N | Y | Y | N | N | N | Y | N | Y | N | 6 |
| Haas et al.2020 | Y | Y | Y | Y | N | Y | N | Y | N | Y | N | 8 |
| Cavazos-Rehg et al.2020 | Y | Y | Y | Y | N | N | N | Y | N | Y | N | 7 |
| Yarhere et al.2020 | Y | Y | N | Y | N | N | N | N | N | Y | N | 5 |
| Rukundo et al.2020 | Y | Y | N | Y | N | N | N | Y | N | Y | N | 6 |
| Pilania R et al.2020 | Y | Y | Y | Y | N | N | N | Y | N | Y | N | 7 |
| Agyemang et al.2020 | Y | Y | Y | Y | N | Y | N | Y | N | Y | N | 8 |
| Kinyanda et al.2019 | Y | Y | N | Y | N | Y | Y | Y | N | Y | N | 8 |
| Kemigisha et al.2019 | Y | Y | Y | Y | N | N | N | Y | N | Y | N | 7 |
| Abebe et al.2019 | Y | Y | Y | Y | N | N | N | Y | N | Y | N | 7 |
| Casale et al.2019 | Y | Y | Y | Y | N | N | Y | Y | N | Y | N | 8 |
| Zhou et al.2019 | Y | Y | N | Y | N | N | Y | Y | N | Y | N | 7 |
| Wonde et al.2019 | Y | Y | Y | Y | N | Y | N | Y | N | Y | N | 8 |
| Coetzee et al.2019 | Y | Y | N | Y | N | Y | N | Y | N | Y | N | 7 |
| Gaitho et al.2018 | Y | Y | Y | Y | N | N | Y | Y | N | Y | N | 8 |
| Ramos et al.2018 | Y | Y | Y | Y | N | N | N | Y | N | Y | N | 7 |
| Study | Response | | | | | | | | | | | |
|  | Q1 | Q2 | Q3 | Q4 | Q5 | Q6 | Q7 | Q8 | Q9 | Q10 | Q11 | Total |
| Okawa et al.2018 | Y | Y | Y | Y | N | Y | N | N | N | Y | N | 7 |
| Lopez et al.2018 | Y | Y | Y | Y | N | N | N | N | N | Y | N | 6 |
| West et al.2018 | Y | Y | Y | Y | N | N | N | Y | N | Y | N | 7 |
| Earnshaw et al.2018 | Y | Y | Y | Y | N | N | N | Y | N | Y | N | 7 |
| Prevost et al.2018 | Y | Y | Y | Y | N | Y | N | Y | N | Y | N | 8 |
| Chenneville et al.2018 | Y | Y | Y | Y | N | N | N | Y | N | Y | N | 7 |
| Woollett et al.2017 | Y | Y | N | Y | N | N | N | Y | N | Y | N | 6 |
| Gentz et al.2017 | Y | Y | Y | Y | N | N | N | Y | N | Y | N | 7 |
| Bankole et al.2017 | Y | Y | N | Y | N | N | N | N | N | Y | N | 5 |
| Dow et al.2016 | Y | Y | Y | Y | N | Y | N | N | Y | Y | N | 8 |
| Fawzi et al.2016 | Y | Y | N | Y | N | Y | N | N | Y | Y | N | 7 |
| Funck Brentano et al.2016 | Y | Y | Y | Y | N | N | N | N | N | Y | N | 6 |
| Côté.J et al.2016 | Y | Y | Y | Y | N | N | N | Y | N | Y | N | 7 |
| Vreeman et al.2015 | Y | N | Y | Y | N | N | N | Y | N | Y | N | 6 |
| Lauren C et al.2015 | Y | Y | Y | Y | N | N | Y | Y | N | Y | N | 8 |
| Lewis et al.2015 | Y | N | N | Y | N | N | N | Y | N | Y | N | 5 |
| Brown et al.2015 | Y | Y | Y | Y | N | N | N | Y | N | Y | N | 7 |
| Kim et al.2014 | Y | Y | Y | Y | N | N | Y | Y | N | Y | N | 8 |

Q1: Define the source of information (survey, record review)

Q2: List the inclusion and exclusion criteria for exposed and unexposed subjects (cases and controls) or refer to previous publications

Q3: Indicate time period used for identifying patients

Q4: Indicate whether or not subjects were consecutive if not population-based

Q5: Indicate if evaluators of subjective components of study were masked to other aspects of the status of the participants

Q6: Describe any assessments undertaken for quality assurance purposes (e.g., test/retest of primary outcome measurements)

Q7: Explain any patient exclusion from analysis

Q8: Describe how confounder was assessed and/or controlled

Q9: If applicable, explain how missing data were handled in the analysis

Q10: Summarize patient response rates and completeness of data collection

Q11: Clarify what follow-up, if any, was expected and the percentage of patients for which incomplete data or follow-up was obtained

(Yes/No/Unclear)

**Reference**

1. Sohn AH, Singtoroj T, Chokephaibulkit K, Lumbiganon P, Hansudewechakul R, Gani YM, et al. Long-Term Post-Transition Outcomes of Adolescents and Young Adults Living With Perinatally and Non-perinatally Acquired HIV in Southeast Asia. Journal of Adolescent Health. (2023) 72(3):471–9. doi: 10.1016/j.jadohealth.2022.10.021

2. Olashore AA, Paruk S, Akanni OO, Chiliza B. Psychiatric disorders in adolescents living with HIV in Botswana. AIDS Res Ther. (2023) 20(1):2. doi: 10.1186/s12981-022-00490-z

3. Nguyen N, Lovero KL, Falcao J, Brittain K, Zerbe A, Wilson IB, et al. Mental health and ART adherence among adolescents living with HIV in Mozambique. AIDS Care. (2023) 35(2):182–90. doi: 10.1080/09540121.2022.2032574

4. Ndongo FA, Kana R, Nono MT, Noah JPYA, Ndzie P, Tejiokem MC, et al. Mental health troubles among Cameroonian adolescents perinatally infected with Human Immunodeficiency Virus. Rev Epidemiol Sante Publique. (2023) 71(3):101422. doi: 10.1016/j.respe.2022.101422

5. Mugo C, Kohler P, Kumar M, Badia J, Kibugi J, Wamalwa DC, et al. Effect of HIV stigma on depressive symptoms, treatment adherence, and viral suppression among youth with HIV. AIDS. (2023) 37(5):813–21. doi: 10.1097/QAD.0000000000003473

6. Gamassa E, Steven E, Mtei R, Kaaya S. Prevalence of Depression and Suicidal Ideation and Associated Risk Factors in Adolescents Receiving Care and Treatment for Hiv/Aids at a Tertiary Health Facility in Kilimanjaro Region, Tanzania. Research Square. (2023) rs.3.rs-2534893 doi: 10.21203/rs.3.rs-2534893/v1

7. Brooks M, Burmen B, Olashore A, Gezmu AM, Molebatsi K, Tshume O, et al. Symptoms of depression, anxiety, and thoughts of suicide/self-injury in adolescents and young adults living with HIV in Botswana. African journal of AIDS research. (2023) 22(1):54–62. doi: 10.2989/16085906.2023.2186252

8. Arnold EM, Yalch MM, Christodoulou J, Murphy DA, Swendeman D, Rotheram-Borus MJ, et al. Rumination influences the relationship between trauma and depression over time among youth living with HIV. Journal of affective disorders. (2023) 322:9–14. doi: 10.1016/j.jad.2022.11.010

9. Zhou Y, Tang K, Lu H, Chen H, Xie H, Li Z, et al. Behavioral and emotional difficulties and HIV treatment outcomes among HIV-infected children in rural southwestern China. Child and Adolescent Psychiatry and Mental Health. (2023) 17(1):1–10. doi: 10.1186/s13034-023-00601-2

10. Olashore AA, Paruk S, Tshume O, Chiliza B. Depression and suicidal behavior among adolescents living with HIV in Botswana: a cross-sectional study. Child and Adolescent Psychiatry and Mental Health. (2022) 16(1):62. doi: 10.1186/s13034-022-00492-9

11. Chory A, Callen G, Nyandiko W, Njoroge T, Ashimosi C, Aluoch J, et al. A pilot study of a mobile intervention to support mental health and adherence among adolescents living with HIV in Western Kenya. AIDS and Behavior. (2022) 1–11. doi: 10.1007/s10461-021-03376-9

12. Satriawibawa IW, Wati K, Windiani IG, Adnyana IGA, Vedaswari PD, Sutawan IB. Factors associated with anxiety in children and adolescents with HIV infection. HIV & AIDS Review International Journal of HIV-Related Problems. (2022) 21(2):137–43. doi: 10.5114/hivar.2022.115828

13. Di Gennaro F, Marotta C, Ramirez L, Cardoso H, Alamo C, Cinturao V, et al. High prevalence of mental health disorders in adolescents and youth living with HIV: an observational study from eight health services in Sofala Province, Mozambique. AIDS Patient Care and STDs. (2022) 36(4):123–9. doi: 10.1089/apc.2022.0007 apc.2022.0007

14. Chantaratin S, Trimetha K, Werarak P, Lapphra K, Maleesatharn A, Rungmaitree S, et al. Depression and anxiety in youth and young adults living with HIV: frequency and associated factors in Thai setting. Journal of the International Association of Providers of AIDS Care (JIAPAC). (2022) 21:23259582221101811. doi: 10.1177/23259582221101811

15. Nyongesa MK, Mwangi P, Kinuthia M, Hassan AS, Koot HM, Cuijpers P, et al. Prevalence, risk and protective indicators of common mental disorders among young people living with HIV compared to their uninfected peers from the Kenyan coast: a cross-sectional study. BMC psychiatry. (2021) 21(1):1–17. doi: 10.1186/s12888-021-03079-4

16. Namuli JD, Nalugya JS, Bangirana P, Nakimuli-Mpungu E. Prevalence and factors associated with suicidal ideation among children and adolescents attending a pediatric HIV clinic in Uganda. Frontiers in Sociology. (2021) 6:656739. doi: 10.3389/fsoc.2021.656739

17. Mugo C, Seeh D, Guthrie B, Moreno M, Kumar M, John-Stewart G, et al. Association of experienced and internalized stigma with self-disclosure of HIV status by youth living with HIV. AIDS and Behavior. (2021) 25:2084–93. doi: 10.1007/s10461-020-03137-0

18. Kohn JN, Loop MS, Kim-Chang JJ, Garvie PA, Sleasman JW, Fischer B, et al. Trajectories of depressive symptoms, neurocognitive function and viral suppression with antiretroviral therapy among youth with HIV over 36 months. Journal of acquired immune deficiency syndromes. (2021) 87(2):851-859. doi: 10.1097/QAI.0000000000002653

19. Girma D, Assegid S, Gezahegn Y. Depression and associated factors among HIV-positive youths attending antiretroviral therapy clinics in Jimma town, southwest Ethiopia. PLoS One. (2021) 16(1):e0244879. doi: 10.1371/journal.pone.0244879

20. Getaye A, Cherie N, Bazie GW, Gebremeskel Aragie T. Proportion of depression and its associated factors among youth HIV/AIDS clients attending ART clinic in Dessie town government health facilities, Northeast Ethiopia. Journal of Multidisciplinary Healthcare. (2021) 197–205. doi: 10.2147/JMDH.S296849

21. Filiatreau LM, Pettifor A, Edwards JK, Masilela N, Twine R, Xavier Gómez-Olivé F, et al. Associations between key psychosocial stressors and viral suppression and retention in care among youth with HIV in rural South Africa. AIDS and Behavior. (2021) 25:2358–68. doi: 10.1007/s10461-021-03198-9

22. Aurpibul L, Sophonphan J, Malee K, Kerr SJ, Sun LP, Ounchanum P, et al. HIV-related enacted stigma and increase frequency of depressive symptoms among Thai and Cambodian adolescents and young adults with perinatal HIV. International journal of STD & AIDS. (2021) 32(3):246–56. doi: 10.1177/0956462420960602

23. Ashaba S, Cooper-Vince CE, Maling S, Satinsky EN, Baguma C, Akena D, et al. Childhood trauma, major depressive disorder, suicidality, and the modifying role of social support among adolescents living with HIV in rural Uganda. Journal of affective disorders reports. (2021) 4:100094. doi: 10.1016/j.jadr.2021.100094

24. Adeyemo S, Adeosun II, Ogun OC, Adewuya A, David AN, Adegbohun AA, et al. Depression and suicidality among adolescents living with human immunodeficiency virus in Lagos, Nigeria. Child and Adolescent Psychiatry and Mental Health. (2020) 14:1–10. doi: 10.1186/s13034-020-00337-3

25. Buckley J, Otwombe K, Joyce C, Leshabane G, Hornschuh S, Hlongwane K, et al. Mental health of adolescents in the era of antiretroviral therapy: is there a difference between HIV-infected and uninfected youth in South Africa? Journal of Adolescent Health. (2020) 67(1):76–83. doi: 10.1016/j.jadohealth.2020.01.010

26. Dyer J, Wilson K, Badia J, Agot K, Neary J, Njuguna I, et al. The psychosocial effects of the COVID-19 pandemic on youth living with HIV in Western Kenya. AIDS and Behavior. (2021) 25:68–72. doi: 10.1007/s10461-020-03005-x

27. Ekat MH, Yotebieng M, Leroy V, Mpody C, Diafouka M, Loubaki G, et al. Association between depressive symptoms and adherence among adolescents living with HIV in the Republic of Congo: a cross sectional study. Medicine. (2020) 99(35). doi: 10.1097/MD.0000000000021606

28. Haas AD, Technau KG, Pahad S, Braithwaite K, Madzivhandila M, Sorour G, et al. Mental health, substance use and viral suppression in adolescents receiving ART at a paediatric HIV clinic in South Africa. Journal of the International AIDS Society. (2020) 23(12):e25644. doi: 10.1002/jia2.25644

29. Cavazos-Rehg P, Xu C, Kasson E, Byansi W, Bahar OS, Ssewamala FM. Social and economic equity and family cohesion as potential protective factors from depression among adolescents living with HIV in Uganda. AIDS and Behavior. 2020;24:2546–54. doi: 10.1007/s10461-020-02812-6

30. IE Y. "Beck Depression Inventory scores for children with some chronic diseases (Type I diabetes mellitus, Sickle cell anaemia, and AIDS) on management in University of Port Harcourt Teaching Hospital". African Journal of Diabetes Medicine. (2020) 28(1).

31. Rukundo GZ, Mpango RS, Ssembajjwe W, Gadow KD, Patel V, Kinyanda E. Prevalence and risk factors for youth suicidality among perinatally infected youths living with HIV/AIDS in Uganda: the CHAKA study. Child and adolescent psychiatry and mental health. (2020) 14:1–9. doi: 10.1186/s13034-020-00348-0

32. Pilania R, Hemal A, Agarwal S, Beniwal RP, Arora SK. Psychiatric problems amongst adolescents living with HIV at a tertiary care centre in India. Indian Pediatrics. (2020) 57:1026–8.

33. Agyemang EO, Dapaah JM, Osei FA, Appiah SCY, Mensah NK, Odoom SF, et al. Self-esteem assessment among adolescents living with HIV and seeking healthcare at Komfo Anokye Teaching Hospital-Kumasi, Ghana. Journal of the International Association of Providers of AIDS Care. (2020) 19:2325958220976828. doi: 10.1177/2325958220976828

34. Kinyanda E, Salisbury TT, Levin J, Nakasujja N, Mpango RS, Abbo C, et al. Rates, types and co-occurrence of emotional and behavioural disorders among perinatally HIV-infected youth in Uganda: the CHAKA study. Social psychiatry and psychiatric epidemiology. (2019) 54:415–25. doi: 10.1007/s00127-019-01675-0

35. Kemigisha E, Zanoni B, Bruce K, Menjivar R, Kadengye D, Atwine D, et al. Prevalence of depressive symptoms and associated factors among adolescents living with HIV/AIDS in South Western Uganda. AIDS care. (2019) doi: 10.1080/09540121.2019.1566511

36. Abebe H, Shumet S, Nassir Z, Agidew M, Abebaw D. Prevalence of depressive symptoms and associated factors among HIV-positive youth attending ART follow-up in Addis Ababa, Ethiopia. AIDS research and treatment. (2019) 2019:4610458. doi: 10.1155/2019/4610458

37. Casale M, Boyes M, Pantelic M, Toska E, Cluver L. Suicidal thoughts and behaviour among South African adolescents living with HIV: Can social support buffer the impact of stigma? Journal of affective disorders. (2019) 245:82–90. doi: 10.1016/j.jad.2018.10.102

38. Zhou E, Qiao Z, Cheng Y, Zhou J, Wang W, Zhao M, et al. Factors associated with depression among HIV/AIDS children in China. International Journal of Mental Health Systems. (2019) 13:1–9. doi: 10.1186/s13033-019-0263-1

39. Wonde M, Mulat H, Birhanu A, Biru A, Kassew T, Shumet S. The magnitude of suicidal ideation, attempts and associated factors of HIV positive youth attending ART follow ups at St. Paul’s hospital Millennium Medical College and St. Peter’s specialized hospital, Addis Ababa, Ethiopia, 2018. PloS one. (2019) 14(11):e0224371. doi: 10.1371/journal.pone.0224371

40. Coetzee BJ, Loades ME, Du Toit S, Kagee A. Correlates of fatigue among South African adolescents living with HIV and receiving antiretroviral therapy. AIDS and Behavior. (2019) 23:602–8. doi: 10.1007/s10461-018-02384-6

41. Gaitho D, Kumar M, Wamalwa D, Wambua GN, Nduati R. Understanding mental health difficulties and associated psychosocial outcomes in adolescents in the HIV clinic at Kenyatta National Hospital, Kenya. Annals of general psychiatry. (2018) 17:1–9. doi: 10.1186/s12991-018-0200-8

42. Ramos JV, Mmbaga BT, Turner EL, Rugalabamu LL, Luhanga S, Cunningham CK, et al. Modality of primary HIV disclosure and association with mental health, stigma, and antiretroviral therapy adherence in Tanzanian youth living with HIV. AIDS patient care and STDs. (2018) 32(1):31–7. doi: 10.1089/apc.2017.0196

43. Okawa S, Mwanza Kabaghe S, Mwiya M, Kikuchi K, Jimba M, Kankasa C, et al. Psychological well-being and adherence to antiretroviral therapy among adolescents living with HIV in Zambia. AIDS care. (2018) 30(5):634–42. doi: 10.1080/09540121.2018.1425364

44. López J, Shacham E, Brown T. Suicidal ideation persists among individuals engaged in HIV care in the era of antiretroviral therapy. AIDS and Behavior. (2018) 22:800–5. doi: 10.1007/s10461-016-1666-5

45. West N, Schwartz S, Mudavanhu M, Hanrahan C, France H, Nel J, et al. Mental health in South African adolescents living with HIV. AIDS care. (2019) 31(1):117–24. doi: 10.1080/09540121.2018.1533222

46. Earnshaw VA, Kidman RC, Violari A. Stigma, depression, and substance use problems among perinatally HIV-infected youth in South Africa. AIDS and Behavior. (2018) 22:3892–6. doi: 10.1007/s10461-018-2201-7

47. Le Prevost M, Arenas-Pinto A, Melvin D, Parrott F, Foster C, Ford D, et al. Anxiety and depression symptoms in young people with perinatally acquired HIV and HIV affected young people in England. AIDS care. (2018) 30(8):1040–9. doi: 10.1080/09540121.2018.1441972

48. Chenneville T, Gabbidon K, Lynn C, Rodriguez C. Psychological factors related to resilience and vulnerability among youth with HIV in an integrated care setting. AIDS care. (2018) 30(sup4):5–11. doi: 10.1080/09540121.2018.1488032

49. Woollett N, Cluver L, Bandeira M, Brahmbhatt H. Identifying risks for mental health problems in HIV positive adolescents accessing HIV treatment in Johannesburg. Journal of Child & Adolescent Mental Health. (2017) 29(1):11–26. doi: 10.2989/17280583.2017.1283320

50. Gentz SG, Calonge Romano I, Martínez-Arias R, Ruiz-Casares M. Predictors of mental health problems in adolescents living with HIV in Namibia. Child and Adolescent Mental Health. (2017) 22(4):179–85. doi: 10.1111/camh.12247

51. Bankole KO, Bakare MO, Edet BE, Igwe MN, Ewa AU, Bankole IA, et al. Psychological complications associated with HIV/AIDS infection among children in South-South Nigeria, sub-Saharan Africa. Cogent Medicine. (2017) 4(1):1372869. doi: 10.1080/2331205X.2017.1372869

52. Dow DE, Turner EL, Shayo AM, Mmbaga B, Cunningham CK, O’Donnell K. Evaluating mental health difficulties and associated outcomes among HIV-positive adolescents in Tanzania. AIDS care. (2016) 28(7):825–33. doi: 10.1080/09540121.2016.1139043

53. Smith Fawzi MC, Ng L, Kanyanganzi F, Kirk C, Bizimana J, Cyamatare F, et al. Mental health and antiretroviral adherence among youth living with HIV in Rwanda. Pediatrics. (2016) 138(4). doi: 10.1542/peds.2015-3235

54. Funck-Brentano I, Assoumou L, Veber F, Moshous D, Frange P, Blanche S. Resilience and life expectations of perinatally HIV-1 infected adolescents in France. The Open AIDS Journal. (2016) 10:209. doi: 10.2174/1874613601610010209

55. Côté J, Delmas P, de Menezes Succi RC, Galano E, Auger P, Sylvain H, et al. Predictors and evolution of antiretroviral therapy adherence among perinatally HIV-infected adolescents in Brazil. Journal of Adolescent Health. (2016) 59(3):305–10. doi: 10.1016/j.jadohealth.2016.05.004

56. Vreeman RC, Scanlon ML, Marete I, Mwangi A, Inui TS, McAteer CI, et al. Characteristics of HIV-infected adolescents enrolled in a disclosure intervention trial in western Kenya. AIDS care. (2015) 27(sup1):6–17. doi: 10.1080/09540121.2015.1026307

57. Ng LC, Kirk CM, Kanyanganzi F, Fawzi MCS, Sezibera V, Shema E, et al. Risk and protective factors for suicidal ideation and behaviour in Rwandan children. The British Journal of Psychiatry. (2015) 207(3):262–8. doi: 10.1192/bjp.bp.114.154591

58. V. Lewis J, Abramowitz S, J. Koenig L, Chandwani S, Orban L. Negative life events and depression in adolescents with HIV: a stress and coping analysis. AIDS care. (2015) 27(10):1265–74. doi: 10.1080/09540121.2015.1050984

59. Brown LK, Whiteley L, Harper GW, Nichols S, Nieves A, Interventions A 086 PT for TAMTN for H. Psychological symptoms among 2032 youth living with HIV: a multisite study. AIDS patient care and STDs. (2015) 29(4):212–9. doi: 10.1089/apc.2014.0113

60. Kim MH, Mazenga AC, Devandra A, Ahmed S, Kazembe PN, Yu X, et al. Prevalence of depression and validation of the Beck Depression Inventory-II and the Children’s Depression Inventory-Short amongst HIV-positive adolescents in Malawi. Journal of the International AIDS Society. (2014) 17(1):18965. doi: 10.7448/IAS.17.1.18965
