# Supplementary material for: Prevalence of mental disorders among young people living with HIV: a systematic review and meta-analysis
Source: Front Public Health. 2024 Aug 21;12:1392872. doi: 10.3389/fpubh.2024.1392872 (PMC11372585; doi:10.3389/fpubh.2024.1392872)

**Table and Figure legends**

**Table 1.** Details of search strategy used in this study

**Figure 1.** Forest plot of pooled depression prevalence

**Figure 2.** Forest plot of pooled anxiety prevalence

**Figure 3.** Forest plot of sensitivity analysis for pooled depression prevalence

**Figure 4.** Forest plot of sensitivity analysis for pooled anxiety prevalence

**Figure 5.** Forest plot of pooled suicidal ideation prevalence

**Figure 6.** Forest plot of sensitivity analysis for pooled suicidal ideation prevalence

**Figure 7.** Forest plot of pooled suicidal attempts prevalence

**Figure 8.** Forest plot of pooled lifetime suicidal ideation prevalence

**Figure 9.** Forest plot of pooled lifetime suicidal attempts prevalence

**Figure 10.** Forest plot of pooled PTSD prevalence

**Figure 11.** Forest plot of pooled ADHD prevalence

**Figure 12**. Funnel plot of pooled depression prevalence

**Figure 13**. Funnel plot of pooled anxiety prevalence

**Figure 14.** Funnel plot of pooled suicidal ideation prevalence

**Table 1.** Details of search strategy used in this study

| **PUBMED** | (((("HIV"[Mesh]) OR (((Human Immunodeficiency virus*[Title/Abstract]) OR (AIDS virus*[Title/Abstract])) OR (HIV[Title/Abstract]))) OR (("Acquired Immunodeficiency Syndrome"[Mesh]) OR ((((Acquired Immunodeficiency Syndrome[Title/Abstract]) OR (Acquired Immune Deficiency Syndrome[Title/Abstract])) OR (AIDS[Title/Abstract])) OR (HIV/AIDS[Title/Abstract])))) AND ((("Child"[Mesh]) OR (children[Title/Abstract])) OR (("Adolescent"[Mesh]) OR ((((((((adolescen*[Title/Abstract]) OR (Youth*[Title/Abstract])) OR (Teen*[Title/Abstract])) OR (young people[Title/Abstract])) OR (young adults[Title/Abstract])) OR (teenager*[Title/Abstract])) OR (adolescence[Title/Abstract])) OR (juvenile[Title/Abstract]))))) AND ((((((((("Mental Health"[Mesh]) OR ("Mental Disorders"[Mesh])) OR ((((((((((((((mental health[Title/Abstract]) OR (mental health outcome[Title/Abstract])) OR (mental health impact[Title/Abstract])) OR (mental illness*[Title/Abstract])) OR (mental disorder*[Title/Abstract])) OR (psychiatric illness*[Title/Abstract])) OR (psychiatric disease*[Title/Abstract])) OR (psychiatric disorder*[Title/Abstract])) OR (behavior disorder*[Title/Abstract])) OR (bebavioral problem*[Title/Abstract])) OR (psychological problem*[Title/Abstract])) OR (psychological disorder*[Title/Abstract])) OR (emotional problem*[Title/Abstract])) OR (emotional disorder*[Title/Abstract]))) OR ((("Depression"[Mesh]) OR ("Depressive Disorder"[Mesh])) OR ((((((((((depress*[Title/Abstract]) OR (depressed[Title/Abstract])) OR (depression*[Title/Abstract])) OR (major depression[Title/Abstract])) OR (major depression disorder[Title/Abstract])) OR (depressive symptom*[Title/Abstract])) OR (emotional depression[Title/Abstract])) OR (depressive Neuroses[Title/Abstract]))) OR (Depressive Disorder*[Title/Abstract])))) OR (("Anxiety"[Mesh]) OR (((((((((angst[Title/Abstract])) OR (nervousness[Title/Abstract])) OR (anxiousness[Title/Abstract])) OR (social anxiety[Title/Abstract])) OR (anxiety[Title/Abstract])) OR (social anxieties[Title/Abstract])) OR (anxiety symptom*[Title/Abstract])) OR (anxiety disorder*[Title/Abstract])))) OR (("Suicide"[Mesh]) OR (((((sicid*[Title/Abstract]) OR (suicidal ideation[Title/Abstract])) OR (suicidal thoughts[Title/Abstract])) OR (suicidal behavior*[Title/Abstract])) OR (suicidal behaviour*[Title/Abstract])))) OR (("Self-Injurious Behavior"[Mesh]) OR ((((((self-injury[Title/Abstract]) OR (non-suicidal self-injury[Title/Abstract])) OR (self-harm[Title/Abstract])) OR (self-harming behavior*[Title/Abstract])) OR (self-harming behaviour*[Title/Abstract])) OR (Intentional Self Injur*[Title/Abstract])))) OR (("Stress Disorders, Post-Traumatic"[Mesh]) OR ((Post-Traumatic Stress Disorder[Title/Abstract]) OR (PTSD[Title/Abstract])))) OR (("Attention Deficit Disorder with Hyperactivity"[Mesh]) OR ((Attention Deficit Disorders with Hyperactivity[Title/Abstract]) OR (ADHD[Title/Abstract])))) |
| --- | --- |
| **Cochrane**  **library** | ((HIV[Mesh] OR (HIV OR AIDS virus* OR Human Immunodeficiency virus*):ti,ab,kw)) OR ((Acquired Immunodeficiency Syndrome[Mesh] OR (Acquired Immunodeficiency Syndrome OR Acquired Immune Deficiency Syndrome OR AIDS):ti,ab,kw)) AND ((Child[Mesh] OR Adolescent[Mesh] OR child OR children OR adolescen* OR Youth* OR Teen* OR young people OR young adults or teenager* OR adolescence OR juvenile):ti,ab,kw)) AND ((( Mental Health[Mesh] OR Mental Disorders[Mesh] OR (mental health OR mental health outcome OR mental health impact or mental illness* OR mental disorder* OR psychiatric illness* OR psychiatric disease* OR psychiatric disorder* OR behavior disorder* OR bebavioral problem* OR psychological problem* OR psychological disorder* OR emotional problem* or emotional disorder*):ti,ab,kw)) OR ((Depression[Mesh] OR Depressive Disorder[Mesh] OR (depress* or depressed or depression* or major depression or major depression disorder or depressive symptom* or emotional depression or depressive Neuroses or Depressive Disorder*):ti,ab,kw)) OR (( Anxiety[Mesh] OR (angst or nervousness or anxiousness or social anxiety or anxiety or social anxieties or anxiety symptom* or anxiety disorder*):ti,ab,kw)) OR (( Suicide[Mesh] OR (sicid* or suicidal ideation or suicidal thoughts or suicidal behavior* or suicidal behaviour*):ti,ab,kw)) OR (( Self-Injurious Behavior[Mesh] OR (self-injury or non-suicidal self-injury or self-harm or self-harming behavior* or self-harming behaviour* or Intentional Self Injur*) :ti,ab,kw)) OR (( Stress Disorders, Post-Traumatic[Mesh] OR (Post-Traumatic Stress Disorder or PTSD):ti,ab,kw)) OR (( Attention Deficit Disorder with Hyperactivity[Mesh] OR (Attention Deficit Disorders with Hyperactivity or ADHD):ti,ab,kw))) |
| **Embase** | ('human immunodeficiency virus'/exp OR hiv:ab,ti OR 'aids virus':ab,ti OR 'human immunodeficiency virus':ab,ti OR 'acquired immune deficiency syndrome'/exp OR 'acquired human immunodeficiency syndrome':ab,ti OR aids:ab,ti OR 'acquired immunodeficiency syndrome':ab,ti) AND ( 'child'/exp OR children:ab,ti OR child:ab,ti OR 'adolescent'/exp OR adolescen*:ab,ti OR youth*:ab,ti OR teen*:ab,ti OR 'young people':ab,ti OR 'young adults':ab,ti OR teenager*:ab,ti OR adolescence:ab,ti OR juvenile:ab,ti) AND ( 'mental health'/exp OR 'mental disease'/exp OR 'mental health':ab,ti OR 'mental health outcome':ab,ti OR 'mental health impact':ab,ti OR 'mental illness*':ab,ti OR 'mental disorder*':ab,ti OR 'psychiatric illness*':ab,ti OR 'psychiatric disease*':ab,ti OR 'psychiatric disorder*':ab,ti OR 'behavior disorder*':ab,ti OR 'bebavioral problem*':ab,ti OR 'psychological problem*':ab,ti OR 'psychological disorder*':ab,ti OR 'emotional problem*':ab,ti OR 'emotional disorder*':ab,ti OR 'depression'/exp OR 'major depression'/exp OR depression*:ab,ti OR depress*:ab,ti OR depressed:ab,ti OR 'depressive disorder*':ab,ti OR 'depressive symptom*':ab,ti OR 'depressive illness*':ab,ti OR 'depressive disease*':ab,ti OR 'major depression disorder':ab,ti OR 'anxiety'/exp OR angst:ab,ti OR nervousness:ab,ti OR anxiousness:ab,ti OR 'social anxiety':ab,ti OR anxiety:ab,ti OR 'social anxieties':ab,ti OR 'anxiety symptom*':ab,ti OR 'anxiety disorder*':ab,ti OR 'suicide'/exp OR suicide:ab,ti OR 'suicidal death':ab,ti OR 'self killing':ab,ti OR 'suicidal ideation':ab,ti OR 'suicidal thoughts':ab,ti OR 'suicidal behaviors':ab,ti OR 'automutilation'/exp OR 'self injury':ab,ti OR 'non-suicidal self-injury':ab,ti OR 'self harm':ab,ti OR 'self-harming behavior*':ab,ti OR 'self-injurious behavior*':ab,ti OR 'self harming':ab,ti OR 'posttraumatic stress disorder'/exp OR 'post-traumatic stress disorder':ab,ti OR ptsd:ab,ti OR 'attention deficit hyperactivity disorder'/exp OR 'attention deficit disorders with hyperactivity':ab,ti OR adhd:ab,ti) |

**Figure 1.** Forest plot of pooled depression prevalence


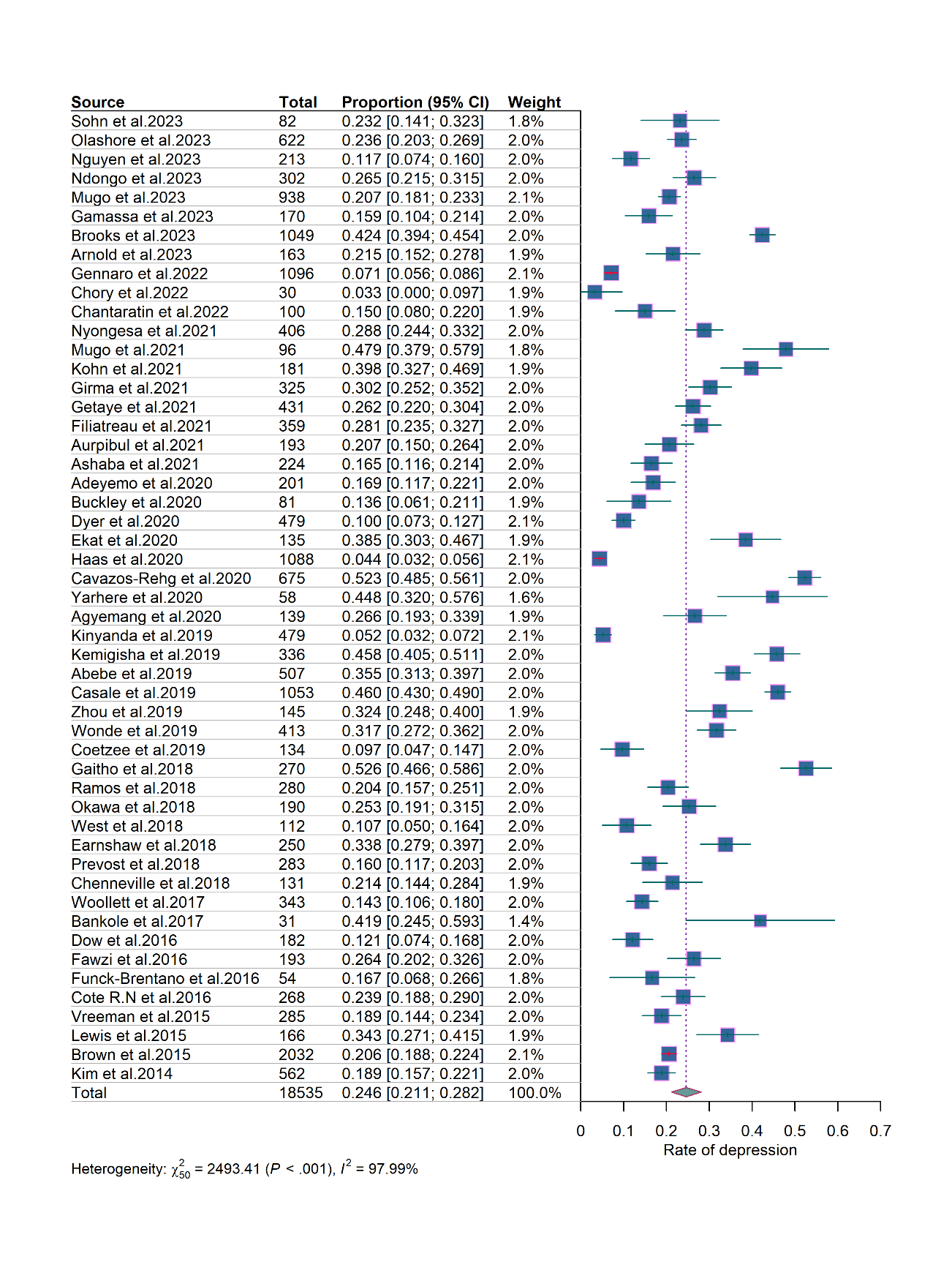


**Figure 2.** Forest plot of pooled anxiety prevalence

**
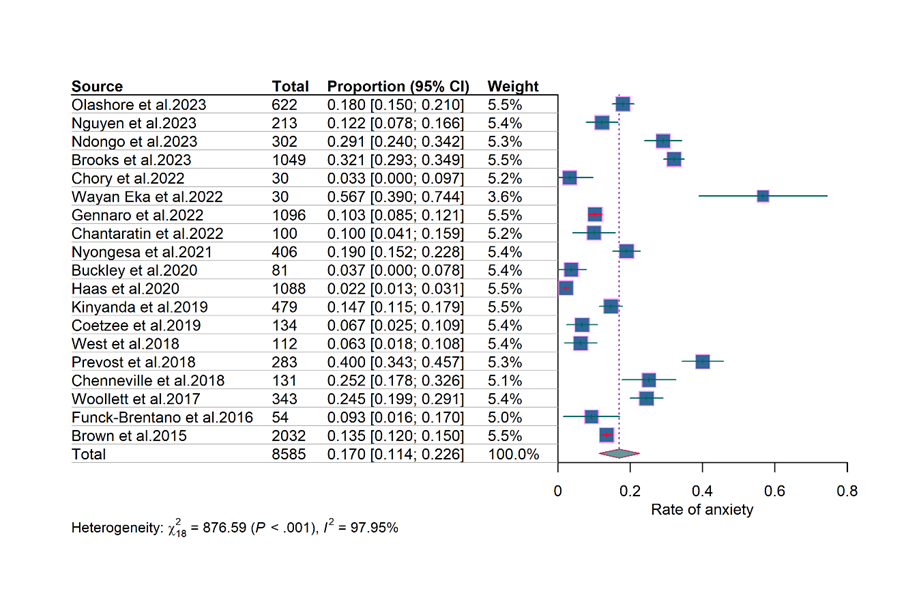
**

**Figure 3.** Forest plot of sensitivity analysis for pooled depression prevalence


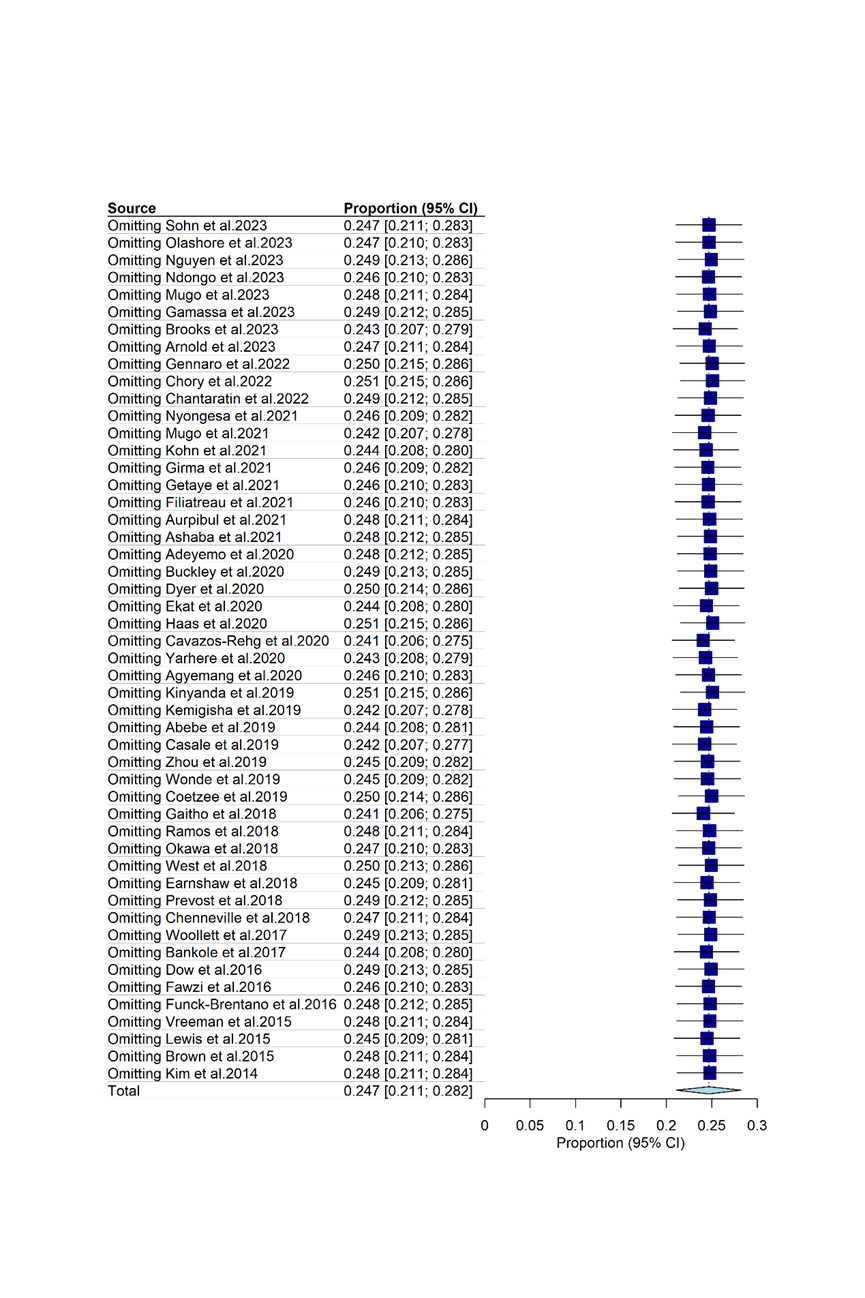


**Figure 4.** Forest plot of sensitivity analysis for pooled anxiety prevalence

**
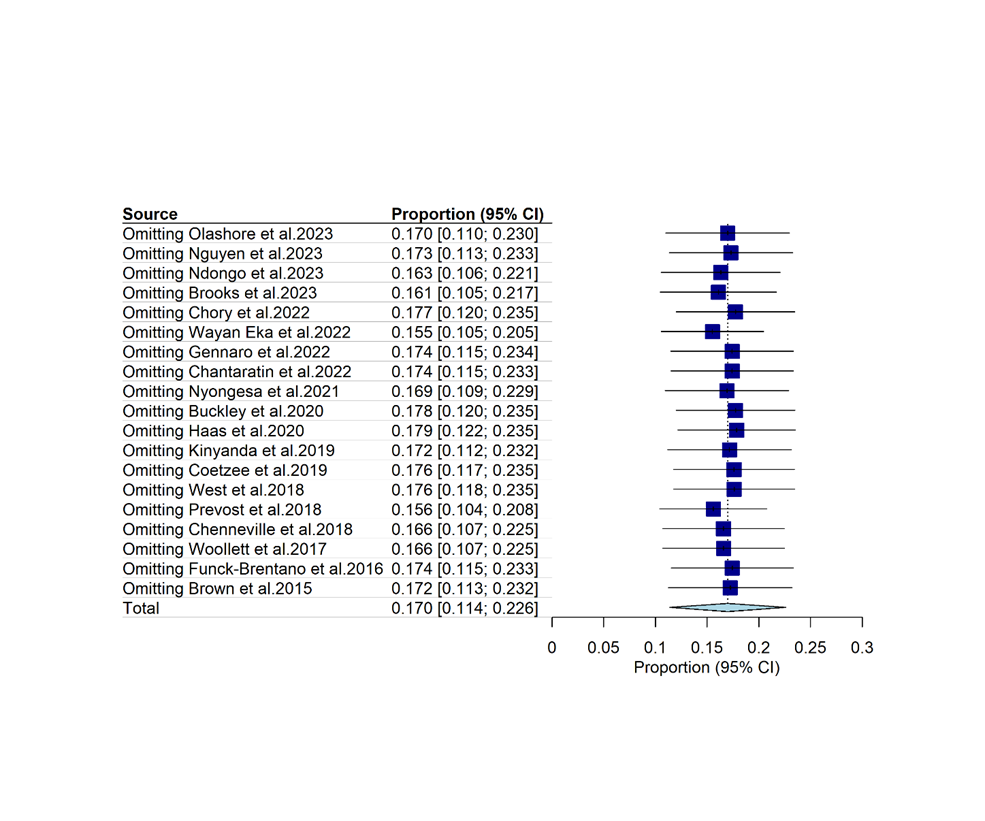
**

**Figure 5.** Forest plot of pooled suicidal ideation prevalence

**
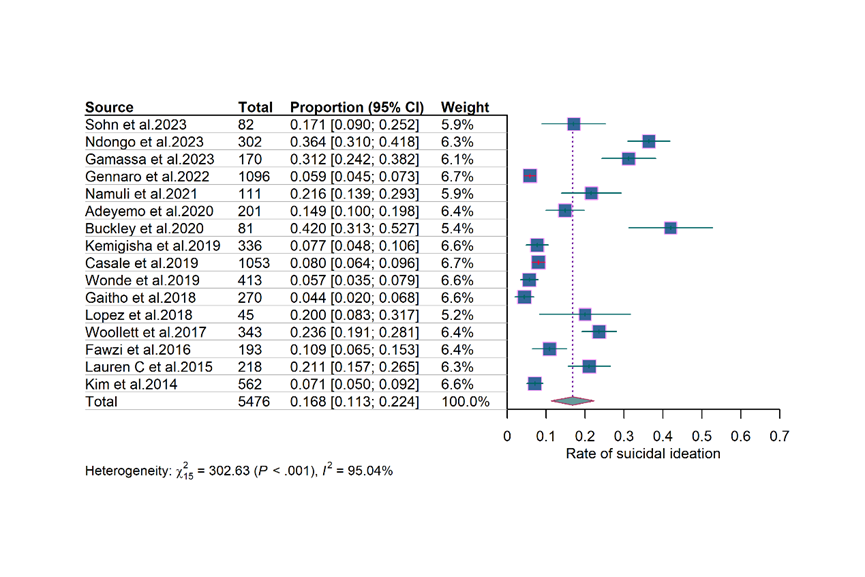
**

**Figure 6.** Forest plot of sensitivity analysis for pooled suicidal ideation prevalence

**
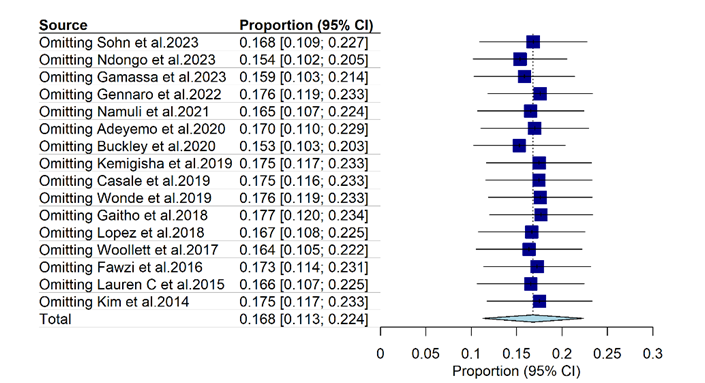
**

**Figure 7.** Forest plot of pooled suicidal attempts prevalence


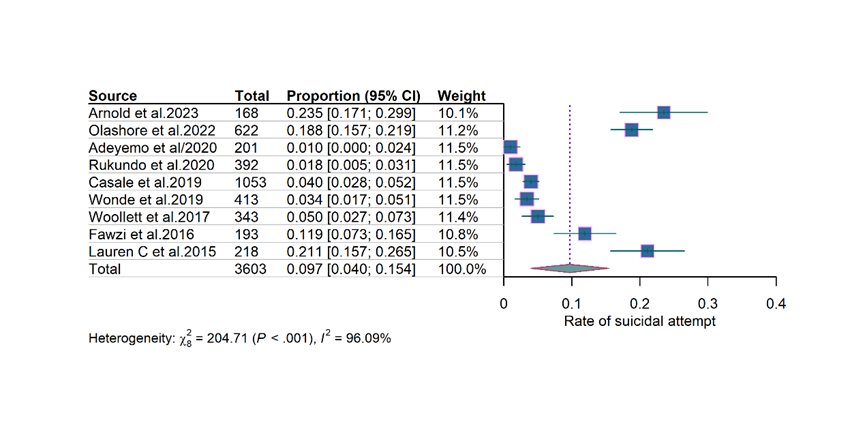


**Figure 8.** Forest plot of pooled lifetime suicidal ideation prevalence


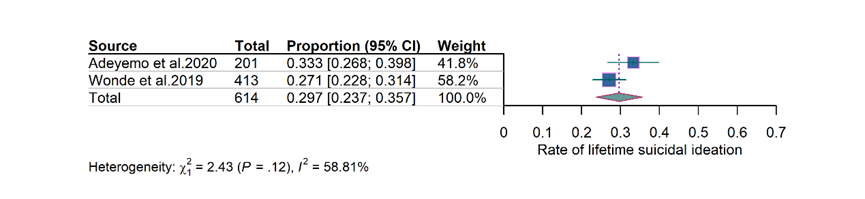


**Figure 9.** Forest plot of pooled lifetime suicidal attempts prevalence


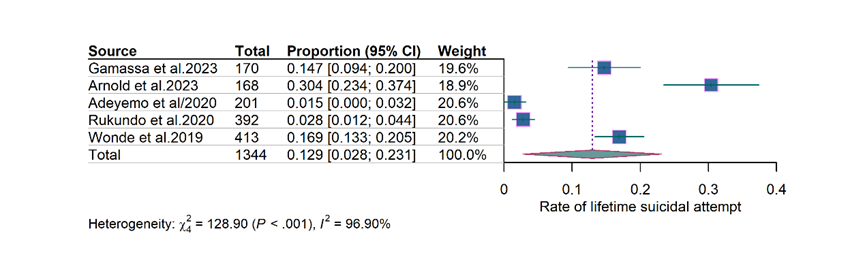


**Figure 10.** Forest plot of pooled PTSD prevalence


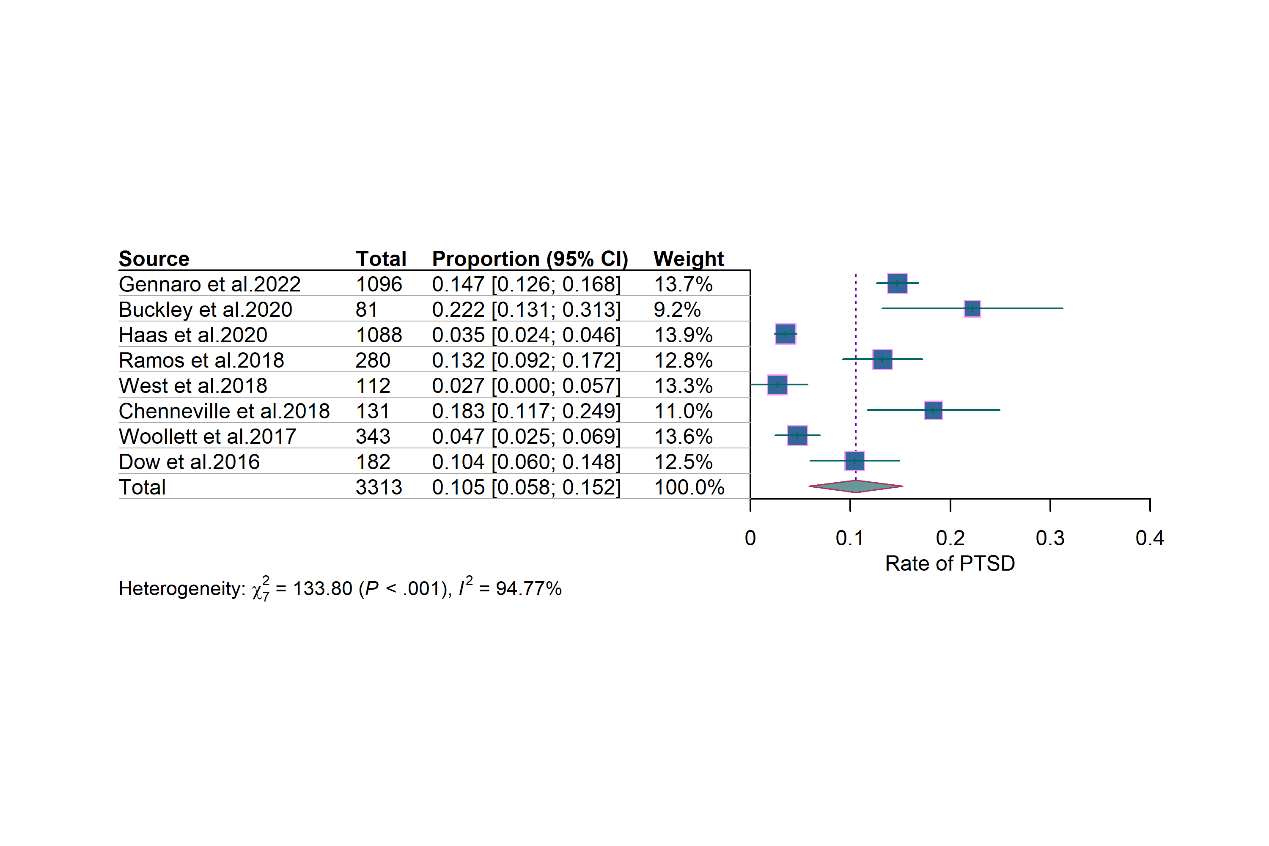


**Figure 11.** Forest plot of pooled ADHD prevalence


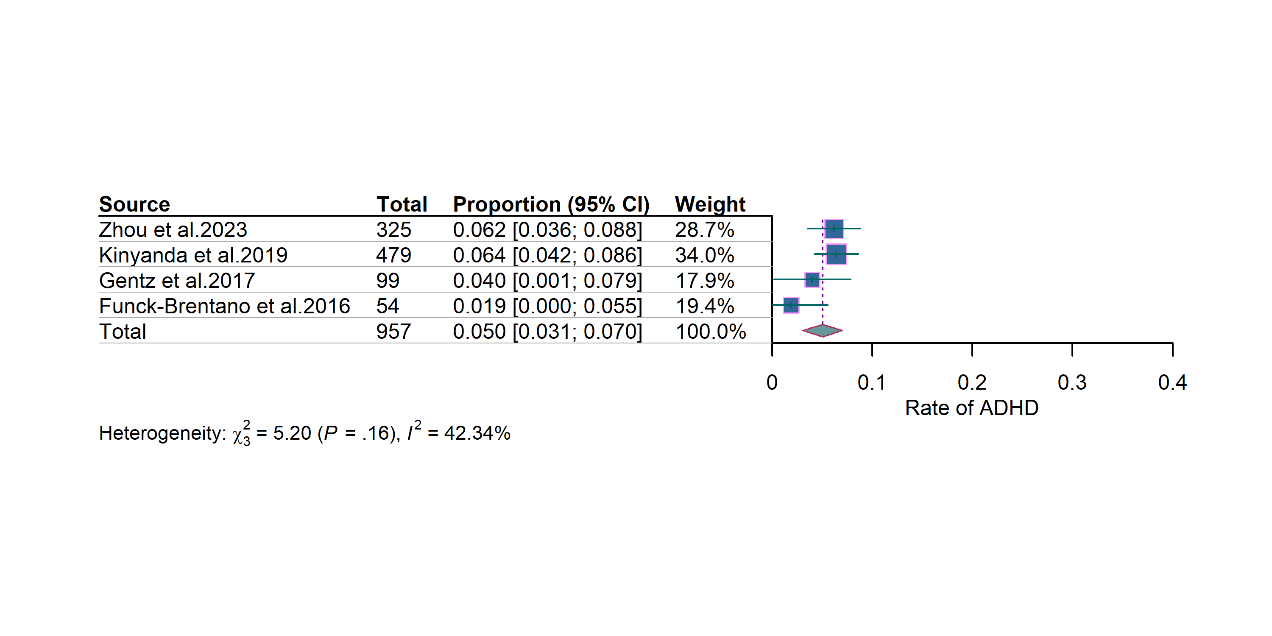


**Figure 12**. Funnel plot of pooled depression prevalence(Egger’s test *P*<0.001)


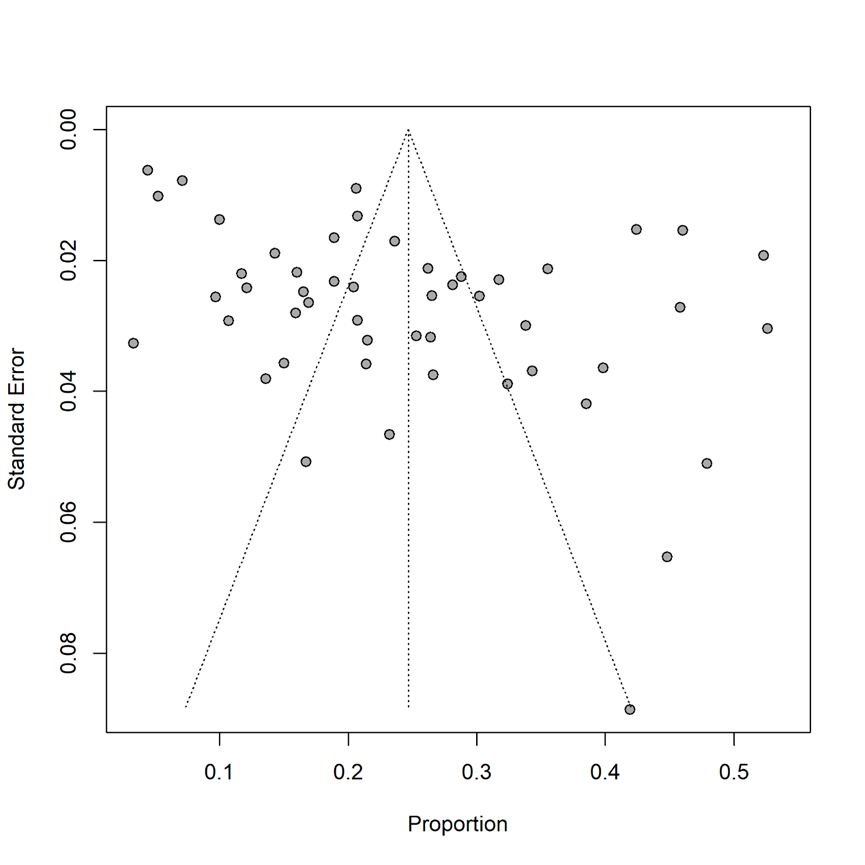


**Figure 13**. Funnel plot of pooled anxiety prevalence(Egger’s test P=0.006)


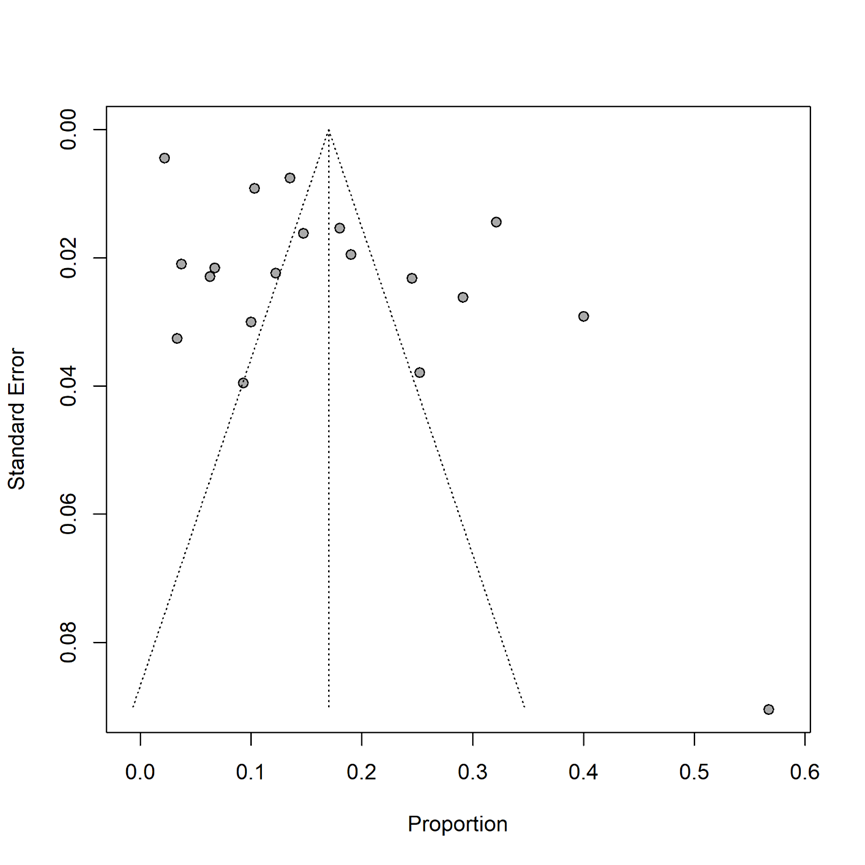


**Figure 14.** Funnel plot of pooled suicidal ideation prevalence(Egger’s test P=0.002)


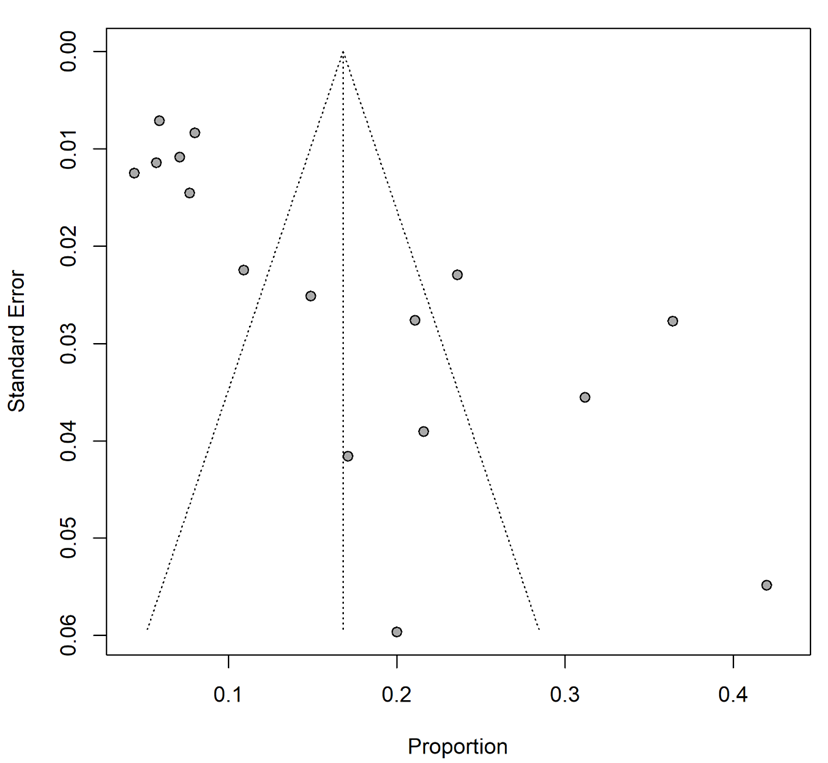

Supplement: Supplementary file 2 [file Data_Sheet_2.docx]
